# Four-dimensional, dynamic mosaicism is a hallmark of normal human skin that permits mapping of the organization and patterning of human epidermis during terminal differentiation

**DOI:** 10.1371/journal.pone.0198011

**Published:** 2018-06-13

**Authors:** Yun Wang, Taro Masaki, Sikandar G. Khan, Deborah Tamura, Christiane Kuschal, Megan Rogers, John J. DiGiovanna, Kenneth H. Kraemer

**Affiliations:** 1 Laboratory of Cancer Biology and Genetics, Center for Cancer Research, National Cancer Institute, National Institutes of Health, Bethesda, MD, United States of America; 2 Department of Dermatology, Peking University First Hospital, Beijing, China; 3 Department of Dermatology, Kobe University School of Medicine, Kobe, Japan; University of Iceland, ICELAND

## Abstract

Recent findings of mosaicism (DNA sequence variation) challenge the dogma that each person has a stable genetic constitution. Copy number variations, point mutations and chromosome abnormalities in normal or diseased tissues have been described. We studied normal skin mosaicism of a single nucleotide polymorphism (SNP) [rs1426654, p.Thr111Ala] in *SLC24A5*, an ion transporter gene. This SNP is unusual in that more than 90% of people of European descent have homozygous germline A/A alleles, while more than 90% of East Asians and Blacks have homozygous germline G/G alleles. We found mosaicism in neonatal foreskins as well as in 69% of nearly 600 skin surface scraping samples from 114 donors of different ages. Strikingly, donors with germline (buccal or blood) A/A, A/G or G/G genotypes had all three sequences (A/A, A/G or G/G) in the skin surface scrapings. SNP sequence differences extended within the epidermis in the vertical dimension from basal cell layer to the stratum corneum at the surface, as well as across the two-dimensions of the skin surface. Furthermore, repeated scrapings in the same location revealed variation in the sequences in the same individuals over time, adding a fourth dimension to this variation. We then used this mosaicism to track the movement of epidermal cells during normal differentiation and characterize the patterning of epidermal cells during terminal differentiation. In this coordinated proliferation model of epidermal differentiation, the skin surface is alternatively populated by synchronous, cycling of waves of cells, with each group having a different DNA sequence. These groups of cells abruptly flatten into large sheets at the surface providing patches of uniform SNP sequence. This four-dimensional mosaicism is a normal, previously unrecognized form of dynamic mosaicism in human skin.

## Introduction

The scope and spectrum of mosaicism in the human is not well characterized. We are becoming increasingly aware of the genetic diversity within the same individual, and how this can be obscure or overt, and dynamically change over space and time. Single nucleotide polymorphisms (SNP) have been thought to be stable genetic markers. In contrast, mosaicism (alterations in the DNA sequence of different areas including chromosome abnormalities, copy number variations, or point mutations) has been reported in benign and malignant neoplasms, and in normal human skin and blood at low frequencies (4% of epidermis [1], 30–40% of blood cells in neurofibromatosis patients [2]; 5% to 31% mutant allele fraction in multiple human tissues [3]; 18–32% of normal skin cells [4]). High frequency benign SNP mosaicism (1000 somatic single nucleotide variants per skin cell) has recently been identified in normal human skin [5].

Mosaicism can occur in the germline, resulting in the possibility of producing progenies with different genotypes that can be subsequently inherited. Post-zygotic mosaicism is seen in a variety of disorders. It can occur early after conception, such as in the nevoid arrays along the skin in the patterning of Blashko’s lines (e.g., mosaic epidermolytic hyperkeratosis [6]). Mosaicism can occur later in development. In cells that retain regenerative potential (i.e., stem cells), localized clones of cells with different genotypes can populate one body location. These can be internally generated (e.g., mosaic pigmentary variations in nevi, café au lait macules, McCune Albright syndrome (reviewed in [6])) or a result of environmentally induced mutation (e.g., UV induced mutations [4]).

The outer layer of the skin is the constantly renewing epidermis, which overlies dermis and subcutaneous fat. Proliferating keratinocytes are confined to the two lowermost epidermal layers and as the basal keratinocytes move up towards the surface, they differentiate and lose the ability to divide. Keratinocytes transit from the basal cell layer to the junction with the stratum corneum, the non-viable, outermost, protective horny layer in about 14 days. Another 14 days are required to travel as sheets through the approximately 20 layers of the stratum corneum (consisting of flattened cells with no nuclei or cell organelles) to the skin surface where they are shed by desquamation [7, 8].

In the autosomal dominant disease, ichthyosis with confetti, a high frequency of spontaneous keratinocyte mosaicism was found in multiple, self-healing areas of epidermis, where somatic loss of disease-causing mutations of *KRT10* or *KRT1* appear as normal islands in a background of congenital erythroderma (red skin)[9, 10]. The keratinocyte clones with normal genotype provide a visual confirmation of proliferating basal keratinocytes migrating to populate the skin surface during cornification. These findings are refocusing the concept of a stable germline towards the realization that organisms are a complex, dynamic genomic mosaic. Similar “revertant mosaicism” has been reported for other hereditary diseases including Bloom syndrome, dyskeratosis congenita, epidermolysis bullosa, Fanconi anemia, tyrosinemia type I, Wiskott-Aldrich syndrome and X-linked severe combined immunodeficiency (reviewed in [11]).

Mosaicism has been used as a tool to map cell migration. Somatic mutations in normal mouse cells have been utilized to reveal developmental lineages of different organs [12]. Similarly, genetic mosaics in humans can provide information about cellular lineages that are difficult to obtain. They can serve as cell markers permitting tracing of development of descendants of the cell in which the new mutation arises (reviewed in [13]). For example, somatic mutations in single human neurons have been used to track developmental history revealing a polyclonal architecture of the human cerebral cortex [14].

We initially investigated pigment variation in the skin of patients with xeroderma pigmentosum, an autosomal recessive disorder due to failure to repair ultraviolet- induced DNA damage which is associated with multiple skin cancers and extensive sunlight-induced, freckle-like, pigmentary abnormalities [15]. We studied a non-synonymous A/G SNP [rs1426654, p.Thr111Ala] in *SLC24A5*, a pigmentation related ion transporter gene. *SLC24A5* is unusual in that more than 90% of people of European descent have homozygous germline A/A alleles, while more than 90% of Blacks and Asians have homozygous germline G/G alleles [16, 17]. Because *SLC24A5* germline sequence is so strongly associated with racial pigmentary differences, we sought to determine if it varied with the pigmentary abnormalities in xeroderma pigmentosum patient’s skin. We were surprised to find a very high frequency of epidermal mosaicism of this SNP, not only in the skin of xeroderma pigmentosum patients, but also in normal appearing skin from donors of different racial groups and different ages from neonatal to adult. We confirmed these results by multiple approaches including Sanger sequencing in both directions, developing an RFLP assay, using a Taq man assay and using a sensitive, allele specific, digital droplet PCR assay. This elevated frequency of SNP sequence variations provides a novel approach for following the patterning of epidermal cells during cornification [18], which we used to develop a better understanding of the migration and patterning of skin epidermal cells during terminal differentiation as a coordinated proliferation model. These findings demonstrate that the surface of normal human skin is dynamically re-populated in layers derived from clusters of cells that share the same DNA sequence.

## Results and discussion

**Fig 1:**
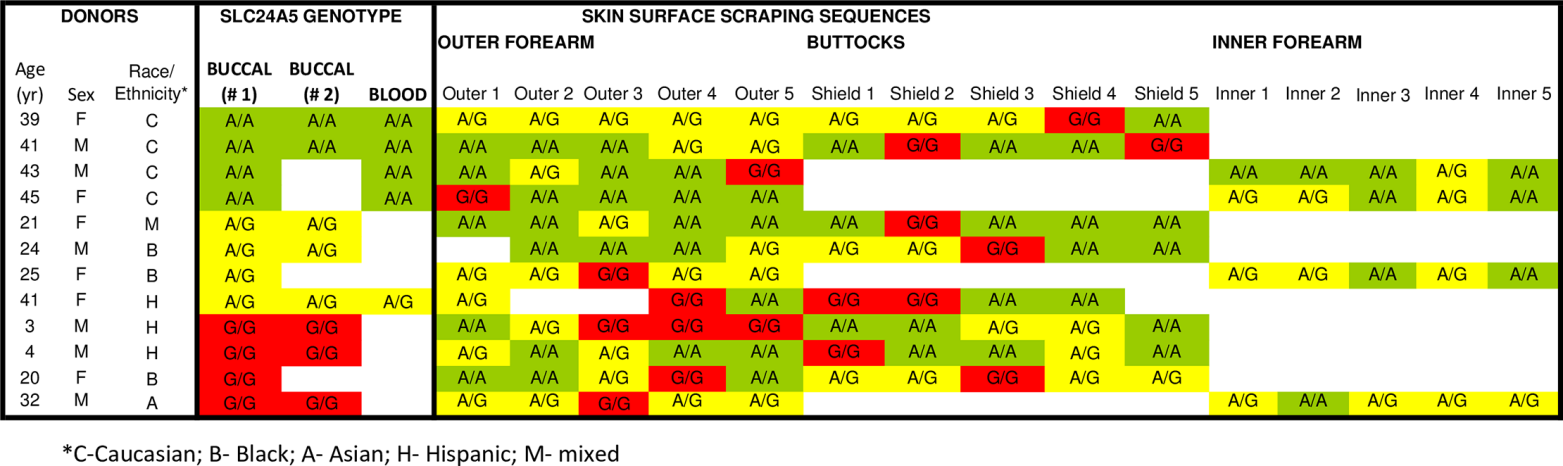
Variation in SNP sequences: Contrast between consistent germline (buccal or blood) cells and the variation in sequences from 116 skin surface scrapings from different anatomic sites in 12 normal donors. DNA from each donor was extracted from separate buccal swabs (left or right sides) and blood and analyzed for presence of *SLC24A5* rs1426654 (A/G) allele. Paired buccal and/or blood samples from the same donor had the same germline genotypes. Multiple skin surface scrapings at different anatomic sites were obtained from normal donors of different ages, genders, and ethnicities. Different SNP sequences were identified at different sites in the same donor. DNA alleles from all samples were determined by Sanger sequencing (see details in Methods).

We studied 114 donors of different racial groups (S1 Table): 86 were clinically normal, 28 had defective DNA repair [15], and 62 (54%) were female. Their *SLC24A5* SNP germline genotype was determined by analyzing DNA from buccal cells and/or blood using restriction fragment length polymorphism (RFLP) assays or Sanger sequencing (S1 Fig). All 59 donors of European descent (Caucasian, White) had homozygous adenine (A) in both alleles: A/A. Twenty-five of the 40 Black or Asian donors had homozygous guanine (G) alleles: G/G. Twenty donors of Black, Asian, Hispanic or Mixed racial groups had a heterozygous A/G genotype. The germline allelic distribution was independent of gender and in agreement with the previously described ethnic distribution [17]. Consistency was evaluated by use of paired blood and/or buccal samples from 86 of the donors (Fig 1 and S2 Table). Within this group, triplicate or quadruplicate samples were independently collected (right and left buccal swabs, and blood) for 31 individuals. The same DNA sequences were identified in each pair for 85 of the donors and only one donor had buccal (G/G) different from blood (A/G) sequences.

### Mosaicism is detectible between different layers of adult skin

**Fig 2:**
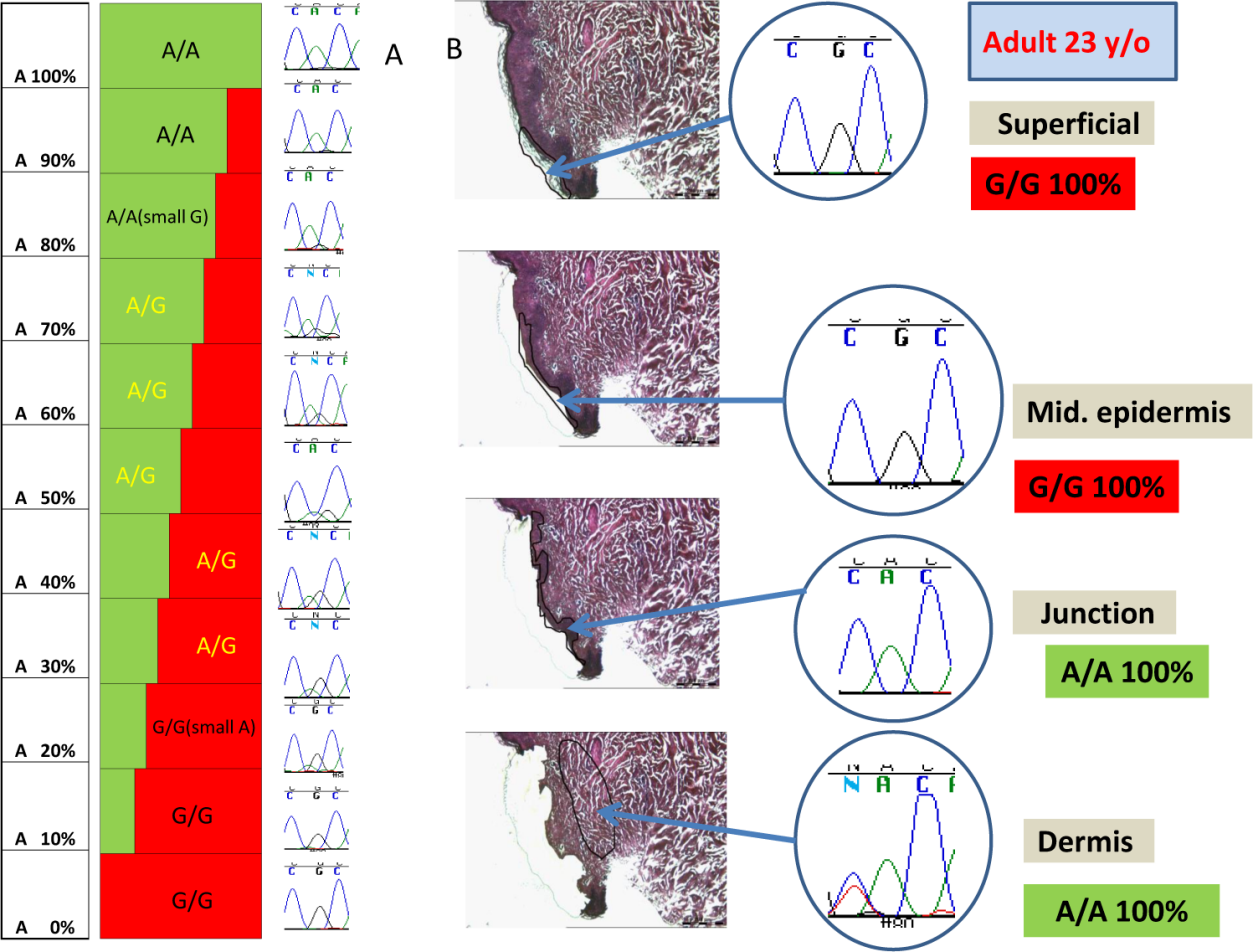
Laser capture microdissection (LCM) and DNA sequencing demonstrating mosaicism between different layers of adult skin. A. Reconstruction experiment, mixing of SLC24A5 A/A and G/G DNA in different proportions followed by Sanger sequencing. The minor allele could be detected at 20–30% of the total DNA. B. LCM and Sanger DNA sequencing of skin of adult with SLC24A5 A/A blood genotype revealed A/A in the epidermal-dermal junction and the dermis and G/G in the superficial and mid epidermis.

Initially, laser capture microdissection (LCM) analysis with Sanger sequencing of a small group of adult skin lesion biopsies (34 biopsies of 7 patients) identified frequent *SLC24A5* sequences that were different from their germline genotype. The epidermis is a constantly renewing epithelial layer, where the lowermost basal cells divide and move vertically towards the surface as they undergo progressive differentiation. We performed LCM of normal skin from an adult (23 yr old) donor with A/A blood germline genotype (Fig 2). As expected, we found A/A sequences at the epidermal/ dermal junction and in the dermis. However, there were G/G sequences in the superficial and mid epidermis. Since the Sanger sequencing technique will only detect sequence differences greater than about 20–30% (Fig 2A), this sequence variation must have been present in a substantial portion of the DNA molecules populating the upper portion of the epidermis. This finding represents mosaicism arrayed within the epidermis in the vertical dimension.

### Early onset of mosaicism in different layers and across the surface of neonatal foreskin

**Fig 3:**
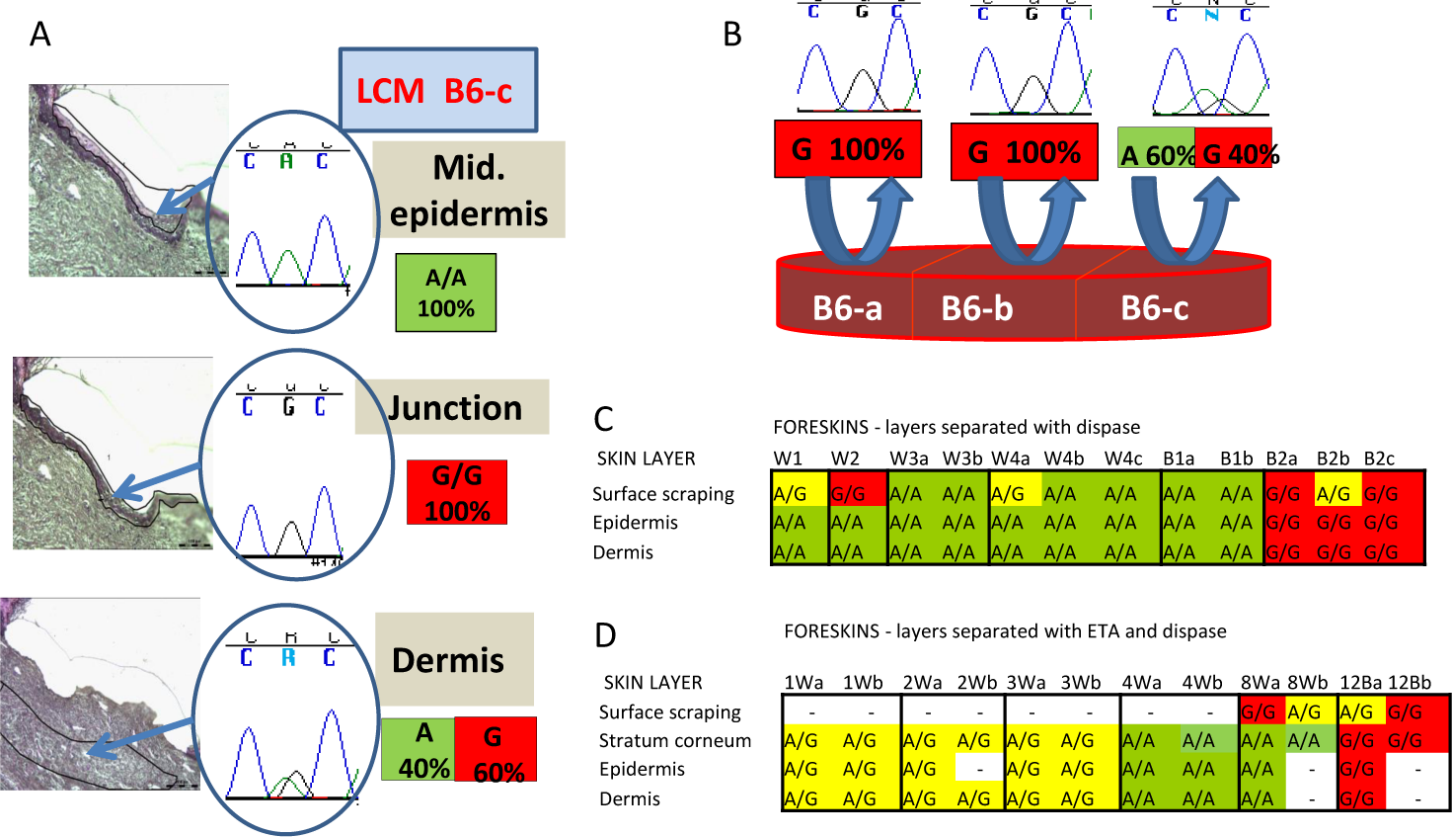
Variation of *SLC24A5* SNP sequences in different layers and across the surface of neonatal foreskins. A. LCM of formalin fixed neonatal foreskin (B6 portion c) followed by DNA sequencing revealed *SLC24A5* A/A in the mid epidermis, G/G at the epidermal/ dermal junction and A/G in the dermis. B. Scraping of the surface of unfixed foreskin B6 (depicted by blue arced arrows) and sequencing the DNA revealed *SLC24A5* G/G in portions a and b and A/G (A 60% and G 40%) in portion c. C. Sequencing of DNA recovered by scraping followed by separation of the epidermis from the dermis with dispase in 6 foreskins. Foreskins W3 and B1 were cut vertically into 2 sections and foreskins W4 and B2 were cut into 3 sections. The surface scrapings of 4 of the 12 sections had different *SLC24A5* alleles than the deeper layers. D. Sequencing of DNA recovered by scraping followed by separation of the stratum corneum with exfoliative toxin A and the epidermis from the dermis with dispase in 6 foreskins. Each foreskin was cut vertically into 2 sections. The surface scrapings of 3 of the sections had regions with different *SLC24A5* alleles than the deeper layers.

To investigate whether these changes had resulted from aging or environmental exposure, we sequenced DNA from anonymized neonatal foreskins obtained shortly after birth (Fig 3). Using LCM to precisely remove small areas (about 300 cells) from different layers of formalin fixed foreskin, we found sequence variations within the same sample: the mid-epidermis was A/A, the junctional epidermis was G/G, and the dermis was G/A (G 60% and A 40% (Fig 3A). The germline genotype was not known for the anonymous foreskins. Similarly, another foreskin revealed A/A sequences in the superficial layers, A/G (A 60% and G 40%) in the mid-epidermis, A/G (A 80% and G 20%) in the epidermal/dermal junction and G/G in the dermis (S2 Fig).

We then examined the surface of the foreskins by gently scraping the outer layer (S3 Fig) and analyzing the DNA. This procedure removed the outermost layers of the stratum corneum for analysis (S4 Fig). We found SNP mosaicism in different surface areas from the same neonatal foreskin (Fig 3B), indicating mosaicism across the skin surface in two horizontal dimensions. For example, foreskin B6 contained two surface regions (B6-a and B6-b) that showed only G sequences and a third region (B6-c) that was A/G (A 60% and G 40%). Some foreskins were further divided into sections and the layers separated enzymatically by use of dispase (which separates the epidermis from the dermis [19]) (S3 Fig) and/or exfoliative toxin A (which separated the upper epidermis from the lower epidermis [20])(S5 Fig). Six of 12 foreskins showed SNP sequence variations in the surface layers compared to the deeper layers (Fig 3C and 3D). This demonstrates mosaicism across the two dimensions of the horizontal surface in addition to the vertical dimension of different layers of skin.

### Confirmation of mosaicism with multiple sequencing methods

**Fig 4:**
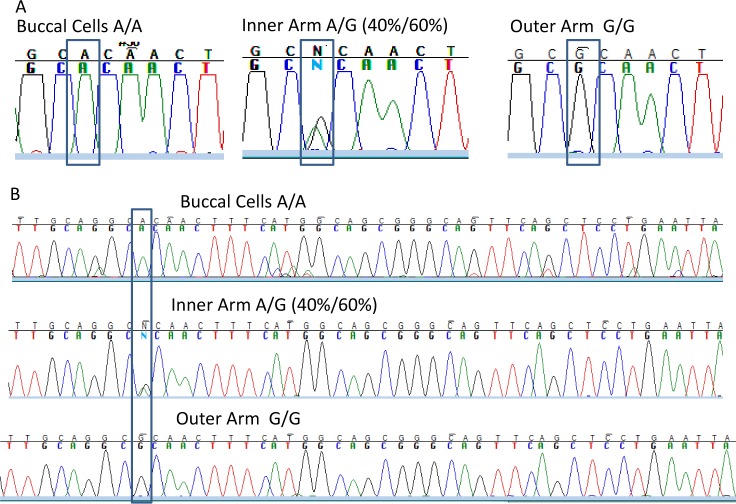
Sanger sequencing of DNA from the buccal cells and 2 skin surface sites from one donor reveal sequence differences. A. Sequencing of nucleotides near the *SLC24A5* SNP site (rectangles). The buccal cells alleles (left) were A/A while the inner arm (middle) was A/G and the outer arm (right) was G/G. B. Approximately 50 nucleotides from the same sequence traces as in A. The only consistent variations were located at the site of the SNP (rectangle).

We developed a minimally invasive technique using skin surface scraping to permit ascertainment of large numbers of samples and repeated sampling over time. After cleaning the skin with 70% isopropyl alcohol, DNA was obtained by gently scraping the skin surface from multiple 25x25 mm squares (S6 Fig). We used Sanger sequencing and two additional assays using different approaches to DNA sequencing to confirm the presence of changes in the *SLC24A5* SNP DNA sequence in the skin scraping samples. Separate cells scraped from the buccal area, and from squares of scraped skin from the inner arm and outer arm of the same individual had A/A, A/G (A 40% and G 60%) or G/G sequences (Fig 4A). Although small variations were present in the Sanger sequence tracings at different locations, the most consistent sequence changes were observed at the *SLC24A5* SNP site (Fig 4B.). We only found A to G or G to A transition sequence changes and did not observe transversions (A to T or C; G to T or C) at this DNA site in any of our more than 600 skin samples assessed by Sanger sequencing.

**Fig 5:**
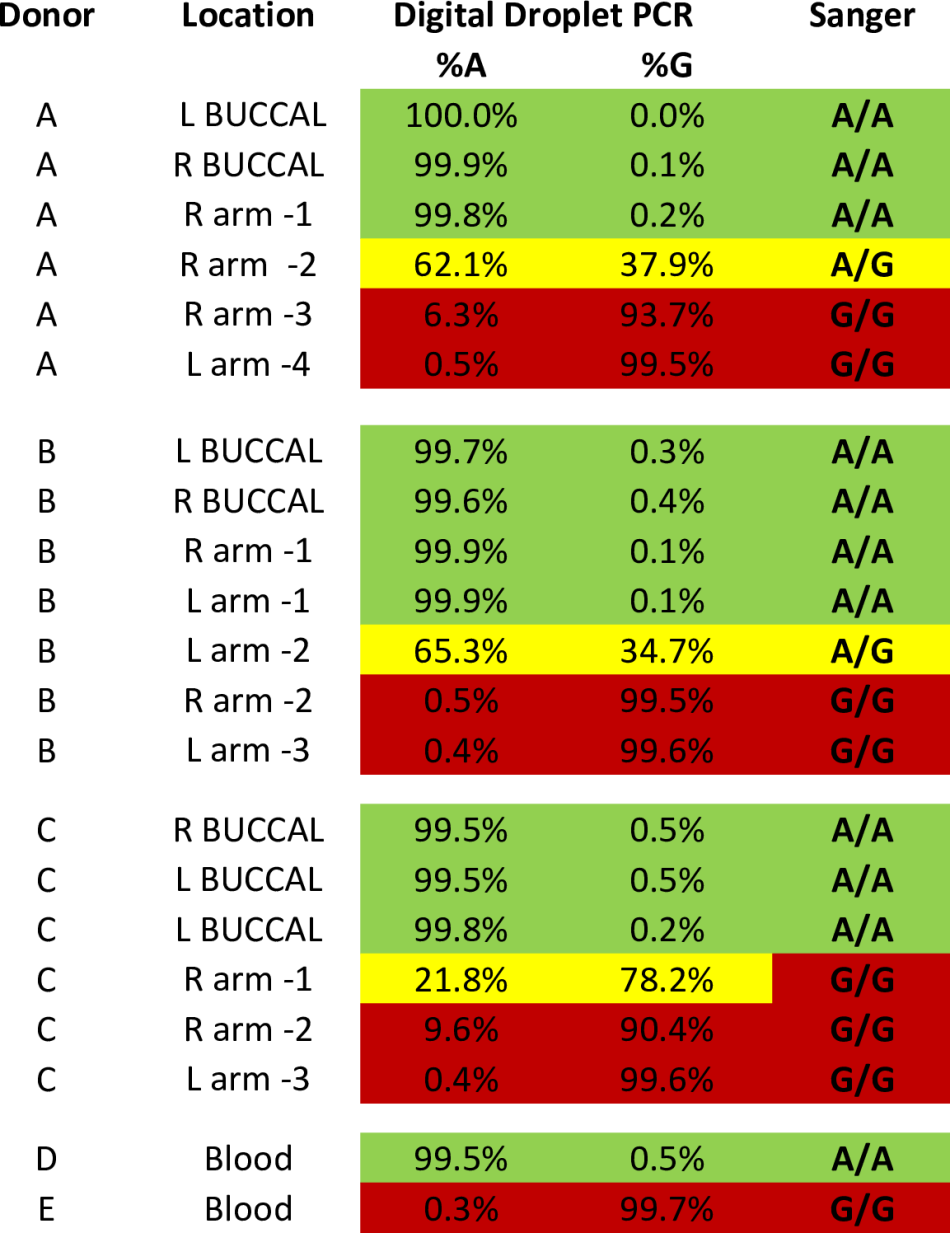
Digital droplet PCR assay of DNA from buccal swabs and skin surface scrapings confirm sequence differences at different anatomic sites. DNA was extracted from buccal swabs (left and right) and from skin surface scrapings from multiple forearm sites from 3 donors. The SLC24A5 alleles were assessed by digital droplet PCR and by Sanger sequencing of the same samples. DNA extracted from blood from donors of known genotype was used as controls. SLC245 allele variation was confirmed by both methods.

We used two additional approaches (TaqMan™ and digital droplet PCR) to confirm the presence of these sequence changes detected by Sanger sequencing in skin scrapings from different sites in a donor. We used TaqMan™ assay with allele specific probes for the A or G allele of the *SLC24A5* SNP (S3 Table). In Donor J the germline buccal DNA was A/A with no detectible G alleles. Three different skin surface scrapings from his arm showed very low levels (4%, 0% and 0%) of G alleles while 5 other scrapings from his arm showed very high levels (99–100%) of G alleles. We then used the very sensitive digital droplet PCR assay that provides quantitative estimates for each allele (Fig 5). Control blood sample with known A/A genotype had less than 1% G while control blood sample with known G/G genotype had less than 1% A. Seven buccal germline samples from 3 donors had greater than 99% A alleles, in agreement with the Sanger sequencing results confirming their germline A/A sequences. Four skin scrapings from Donor A showed different proportions of G alleles (0.2%, 37.9%, 93.7% and 99.5%), in agreement with the Sanger sequencing results. Similar variations in proportion of G alleles (from 0.1% to 99.6%) were noted in skin scrapings from Donors B and C. The digital droplet PCR was more quantitative than the Sanger sequencing in that one sample from Donor C (R arm -1) with 21.8% A was interpreted as G/G by Sanger sequencing. Thus 3 different DNA sequencing assays (Sanger sequencing, Taq Man and digital droplet PCR) confirmed the presence of G alleles in one or more skin scraping samples in donors who had germline homozygosity for the A containing allele of the *SLC24A5* SNP.

### Skin scraping demonstrates a high frequency of mosaicism over the two dimensions of the skin surface area

Three or more skin surface scrapings at different anatomic locations were obtained from 52 donors who had different *SLC24A5* genotypes (A/A, A/G or G/G) for a total of 433 samples. They were analyzed by Sanger sequencing and revealed a high frequency of mosaicism in the *SLC24A5* SNP (Fig 1 and S4 Table). We found variations across different anatomic sites and in both sun shielded and sun exposed skin. The anatomic variations were present in donors of different ages, genders, racial groups and clinical phenotypes.

**Fig 6:**
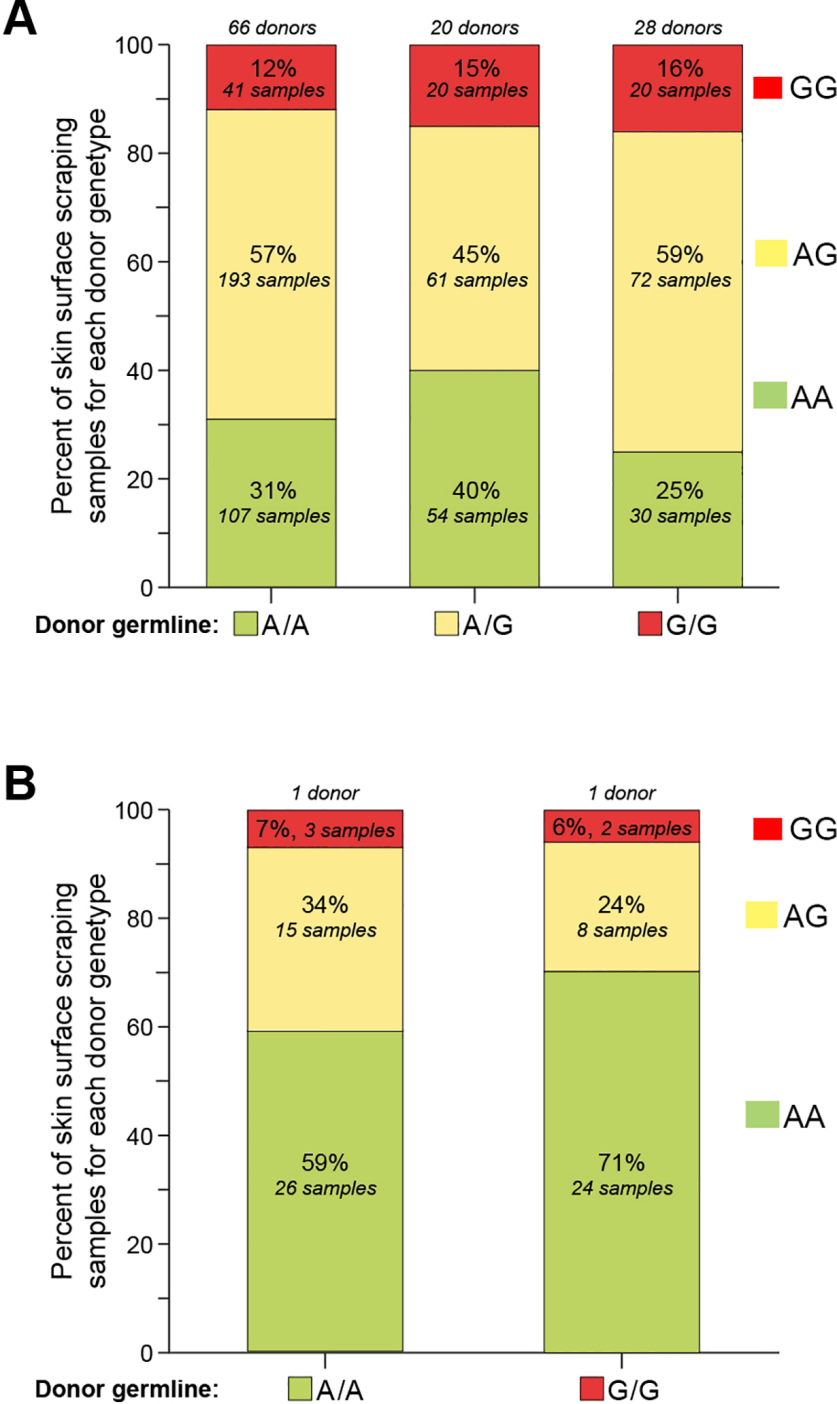
All three sequences (A/A, A/G or G/G) were observed in skin scraping samples of donors with A/A, A/G or G/G germline genotypes. A. Proportion of skin surface scraping samples from 144 donors with *SLC24A5* SNP sequences A/A (green), A/G (yellow) or G/G (red) (each sequence represents a contribution of at least 20–30% of each nucleotide) for each donor of indicated germline genotype (buccal cell or blood). The 66 donors of A/A germline genotype had 341 samples, the 20 A/G germline donors had 135 samples and the 28 G/G germline donors had 122 samples. Multiple samples from each donor were obtained at the same time. B. Proportion of skin surface scraping samples obtained over 91 days from two donors of *SLC24A5* SNP allele germline genotypes A/A (green) or G/G (red) (see Fig 7). The skin surface scraping sequence samples were A/A (green), A/G (yellow) or G/G (red) for each donor of indicated germline genotype. The A/A genotype donor had 44 samples and the G/G genotype donor had 34 samples.

One or two skin surface scrapings were obtained from 62 additional donors for a total of 598 samples from 114 donors (Fig 6). All three sequences (A/A, A/G or G/G) were observed in skin scraping samples of donors with A/A, A/G or G/G germline genotypes and 69% of all sequences were different from the donor's germline genotype (Fig 4A and S5A Table).

Strikingly, donors with homozygous germline (buccal or blood) A/A or G/G genotypes had heterozygous A/G sequences in more than half of the skin surface scrapings. The observation that skin scraping sequences from donors with A/A germline genotype had A/G sequences, and skin scraping sequences from donors with G/G germline genotype also had A/G sequences implies that the sequence variation was not unidirectional (Fig 6 and S5 Table). Donors with homozygous A/A germline alleles had A/G sequences in 57% of their skin surface scraping samples and donors with G/G germline alleles had A/G sequences in 59% of their skin surface scraping samples. Sequences measured at the skin surface are dependent on the mix of cells with each sequence in the sample. A sample measuring A/G could be the result of a surface population composed predominantly of cells harboring the A/G sequence. Alternatively, the same result could be derived from a sample of surface cells containing a mix of cells with A/A and G/G sequences.

**Fig 7:**
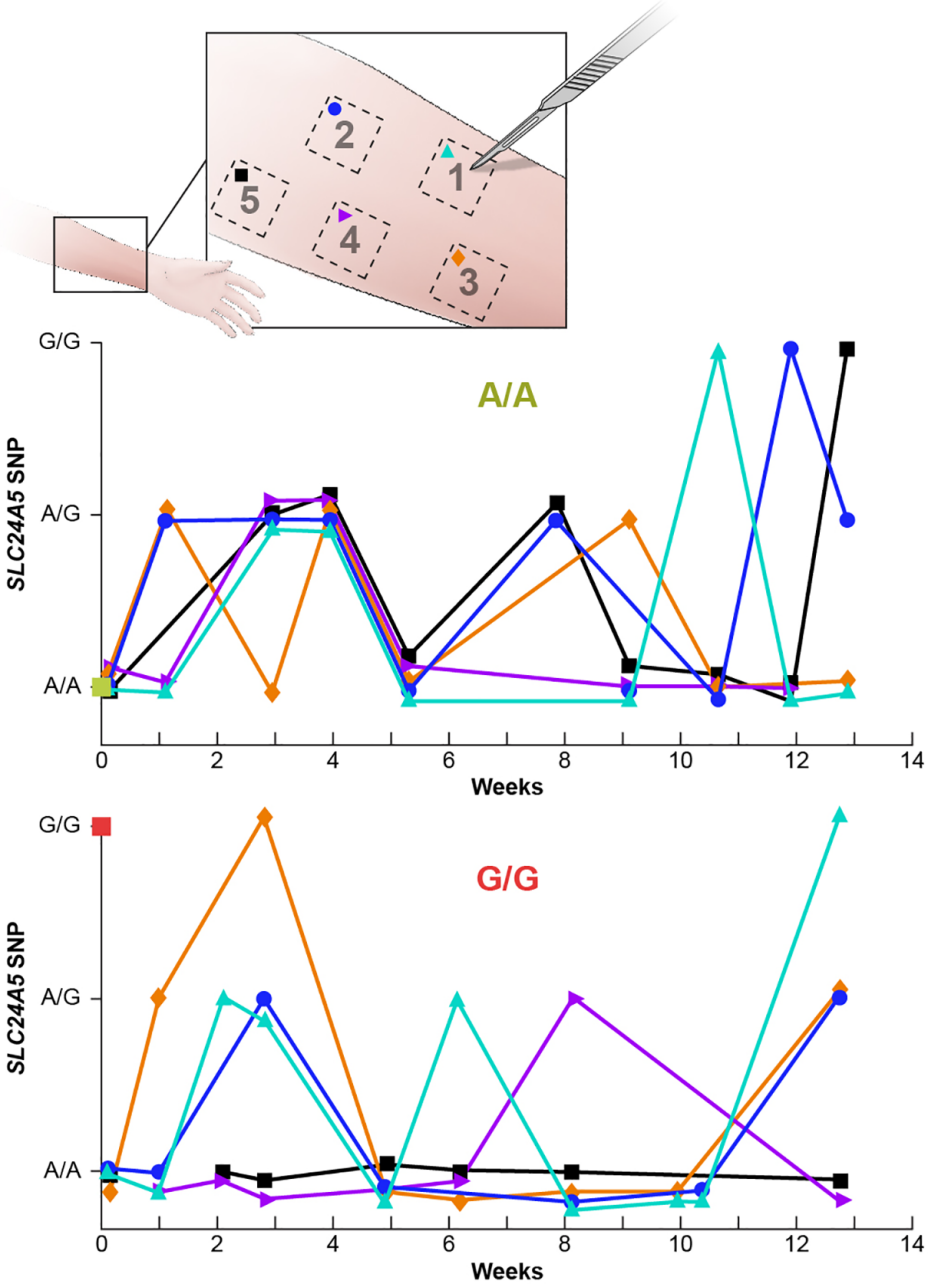
Temporal variation of SLC24A5 SNP sequences in skin surface scrapings from 2 donors. The forearms of two donors were marked with a template indicating five 25 x 25 mm squares (Upper panel). The surface skin in each square was scraped at approximately weekly intervals and the *SLC24A5* sequences determined from the isolated DNA. The buccal cell and blood genotype of the Caucasian donor was A/A (middle panel) and the buccal cell genotype of the Asian donor was G/G (lower panel). The different symbols represent DNA sequences found by scraping each of the squares. The vertical axis indicates A/A or G/G for Sanger sequences that have 20% or less of the minor allele and A/G for all other ratios of allele sequences (see reconstruction experiment Fig 2A).

Surprisingly some of the skin surface scraping samples from donors with homozygous G/G genotype had homozygous A/A sequences (or the reverse) implying a change in two alleles. However, 25% of the G/G germline skin surface scraping samples changed to A/A, and only 12% of the A/A germline changed to G/G [p = 0.002] (Fig 7 and S5 Table).

We hypothesize that the origin of the sequences measured at the skin surface may predate the local epidermal repopulation and arise during embryonic development in the progenitor cells. In this scenario, the sequences variations may originate on a molecular basis. If this difference originates at the molecular level, we would speculate that it may be due to a polymerase bias for the DNA base change made [21–23].

Another possibility is that the sequences measured at the skin surface could result from a differential in the representation of cells with each sequence for example, if A/A cells had a proliferation or migration advantage over G/G. The potential drivers of such differences remain to be determined.

### Identical skin surface locations exhibit dynamic mosaicism that varies over time

To determine whether these SNP variations in *SLC24A5* were present over time, we used a template to ink 5 squares on the normal-appearing forearm skin of two donors (Fig 7 upper panel). We obtained repeated skin surface scraping samples from the same 25x25 mm locations at approximately weekly intervals over 91 days for a total of 78 assessments (Fig 7 and S7 Fig). The germline *SLC24A5* SNP genotype of the Asian donor was G/G while that of the Caucasian donor was A/A. We found that 64% of the samples showed sequence differences from the germline genotype (Fig 6B and S5B Table). Strikingly, there were dynamic alterations in the SNP sequences from the same site over time. Only one of the 10 scraped skin sites in the two donors had the same sequence for the entire 91 days. The other 9 sites all showed SNP sequence variations. Sequence changes were found between samples taken from the same sites as little as 6 to 9 days apart (Fig 7 and S1 Fig). Sequences obtained from the same skin square changed from A/A to A/G or G/G and then back to A/A over 17 to 47 days. Thus, dynamic sequence changes were observed in both directions within the same square of skin surface. Therefore, in addition to variation along the vertical dimension as the skin differentiates, and horizontally across the two dimensions of the skin surface area, this mosaicism varies over time in identical skin locations, signifying four-dimensional mosaicism.

We found variations in 6 other pigmentation related SNPs [24] in adult skin and in foreskins (S8 Fig). These included SNPs located on three different chromosomes (chromosome 15: *MYEF2*, *CTXN2*, *SLC24A5*; chromosome 11: *TYR*; and chromosome 5: *SLC45A2*). In this small set of samples, we observed SNP variations that involved all possible transitions and transversions and occurred in exons, introns and sequences outside of genes. For each SNP Sanger sequencing only identified the two nucleotides indicated and did not find other nucleotides.

### Coordinated proliferation model of epidermal differentiation

We identified a very high frequency of epidermal mosaicism of a non-synonymous A/G SNP in *SLC24A5* in skin from different racial groups and ages (69% of nearly 600 skin surface scraping samples from 114 donors of different ages–S5 Table) and characterized changes in this SNP at the skin surface over time. We used this common, dynamic mosaicism as a tool to identify patterning of epidermal cells as they move to the skin surface during cornification.

Several theories have been proposed to explain how epidermal (interfollicular) basal cells persist while simultaneously producing terminally differentiating keratinocytes including the “epidermal proliferation unit” [25] and “committed progenitor” theories [18, 26–29].

**Fig 8:**
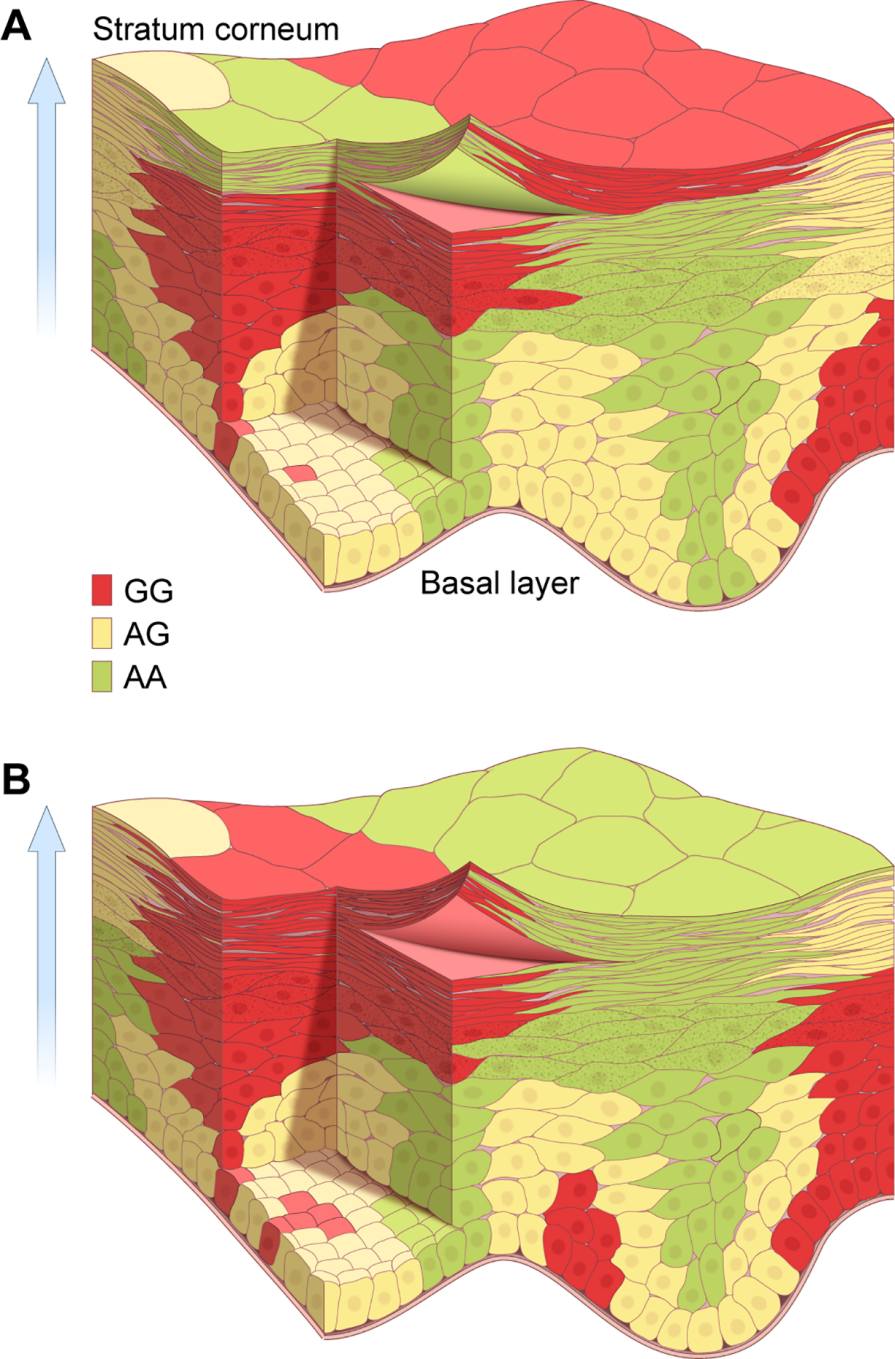
Coordinated proliferation model of epidermal differentiation. This image illustrates the human epidermis from the basal layer to the stratum corneum. The epidermis, a constantly renewing epithelium, is repopulated by division of the lowest-most cells near the basal layer, which move in the vertical dimension (arrow towards the surface) as they differentiate to form corneocytes. Alternating bursts of division at the lowest (basal) layers, result in a surface with patches of cells that have the same sequence. In both panels groups of basal cells with *SCL24A5* sequence of either G/G (red), AG (yellow) or AA (green) proliferate in a burst of activity within the lower two layers of the epidermis. These cells maintain their sequences as they move. As the progeny differentiate, they lose the potential to divide and move vertically towards the surface. They also move laterally, and flatten to occupy a progressively greater surface area. The lateral and vertical movement generates a keratinocyte plume of a uniform sequence with a narrow base that extends over a large area at the surface. At the skin surface, each corneocyte occupies an area 25–35 fold larger than that of their parental basal cell. A. A snapshot of the skin surface when scraping the red area would yield a G/G sequence since most of the surface corneocytes are G/G. As fully differentiated surface corneocytes are shed, the stratum corneum is repopulated with keratinocytes from below. Over time, the G/G containing stem cell would then undergo a quiescent phase similar to the cycling of hair follicles which also contain stem cells. B. Panel B shows the identical skin location at a later time. An adjacent group of basal cells with different *SLC24A5* sequences (A/A, green) has completed a burst of proliferation. Their progeny move into the same skin surface location, populating the surface with a group of corneoctyes with different (A/A) sequences. Skin surface scraping at this time would yield A/A sequences. Depending on the area selected, the scraping could yield A/A, G/G or A/G sequences. This model indicates that a group of proliferative cells with identical genotype produces a clone (coordinated proliferation) of cells that migrate as differentiating keratinocytes not only vertically, but also laterally during their transition to the external surface. This occurs in part because of the large increase in surface area that occurs when the viable stratum granulosum cells near the surface die and form the larger corneocytes at the surface.

Our data is consistent with a coordinated proliferation model of epidermal differentiation where a proliferating cell gives rise to identical daughters with the same SNP sequence that retain the capacity to divide (Fig 8). These cells maintain their sequences as they migrate. In the basal cell layer, keratinocytes have a diameter of 5–6 μm (30 μ^2^ surface area). As they differentiate and move towards the surface, they progressively flatten, a process which has been observed to begin under the stratum corneum (in the stratum granulosum) [30]. At the transition from viable, most superficial stratum granulosum layer to the nonviable stratum corneum, these cells abruptly flatten as they lose their cellular contents and become corneocytes. At the surface they become large, tile-like structures with a surface area of 940–1000 μ^2^ [7, 31, 32]. This patterning has been observed in stem cell marker studies where an umbrella-like surface of progeny is visible from a labelled intrafollicular basal cell (Reference [33]-Fig 7D).

The outermost layer of the 25 x 25 mm skin surface scraping sample contains about 625,000 corneocytes (625 mm^2^ / 1000 μm^2^ per corneocyte). The upper surface area of the corneocyte is 33.3 times that of basal cells (1000 μm^2^ / 30 μm^2^). Thus, the proliferating progenitors of the corneocytes would occupy the equivalent of 18.8 mm^2^ (625 mm^2^ /33.3) at the basal layer [7, 31, 32]. These progeny give rise to clones of differentiating cells that move upwards, flatten and expand to cover a greater surface area, and simultaneously move laterally to create a much larger surface group (patch) of corneocytes with the same sequence (coordinated proliferation) (Fig 8A and 8B).

By serial removal of some of the surface layers in the same surface location (Fig 7) we found variations in the *SLC24A5* SNP sequences over periods as short at 6 to 9 days (S7 Fig). This entails mosaic layering of the stratum corneum since many of the sequence changes were seen at intervals which are shorter than the 14-day stratum corneum turnover time (vertical line in S7 Fig). To convert a major portion of the population of sequences in a skin sample to a new sequence, this model implies a wave of synchronous divisions by proliferating cells with identical SNP sequences (coordinated proliferation) followed by a wave of divisions involving cells with different SNP sequences. Sheets of genetically identical, differentiating keratinocytes appear to move towards the surface in layers of uniform sequence, presumably derived from a related group of proliferative cells. To do so, they must also move laterally. The lateral migration influences the patterning of the skin surface, and is consistent with clinical observations.

The latticework network of rete ridges where the lower layers of the epidermis project into the dermis [34] may provide a means of communication that regulates synchronous cycling of waves of interconnected basal cells–each with a different sequence. A similar pattern of mosaicism has been described for small intestine crypts of mosaic-labelled (Confetti) mice [29, 35].

### Clinical correlates

Previous work demonstrated precancerous mutations in superficial epidermal lesions of actinic keratosis as progeny being derived from proliferating progenitors deeper within the epidermis [36]. While the precancerous pathology originates in the deep stem cells, the clinical lesion only appears at the skin surface when the superficial cells sustain sufficient damage to be unable to form a healthy surface stratum corneum, resulting in a clinically visible lesion (scaling, redness and hyperkeratosis) of actinic keratosis. Similarly, benign warts are clinically identified by their surface appearance. During treatment of either of these types of lesions, if ablation is confined to the visible surface lesion, such as with cryosurgery, the lesion can recur at an edge or nearby. This would result if progenitor cells containing the determining precancerous mutations or human papilloma virus DNA are not situated directly below the superficial clinical lesion. That is, the column of cryosurgical ablation does not destroy the progenitors that are positioned lateral to the surface lesion.

In contrast, the clinically revertant areas of ichthyosis with confetti suggests a different pathophysiology. Ichthyosis with confetti highlights frequent revertant mosaicism, characterized by the development of white, normal appearing islands of skin, with a defined mechanism involving chromosomal translocation [9]. The disease affected areas which cover most of the skin surface have both scaling and redness (erythema). The redness is due to vasodilatation of the dermal vasculature. The upper dermal vasculature may be preferentially influenced by the directly overlying epidermal cells, which are in the lowest epidermal layers. Interestingly, since the cells in the lowest most epidermal layer have little lateral motion it should not be surprising that the borders of the white revertants are stable.

### Reduced DNA replication and repair proteins in skin cells

**Fig 9:**
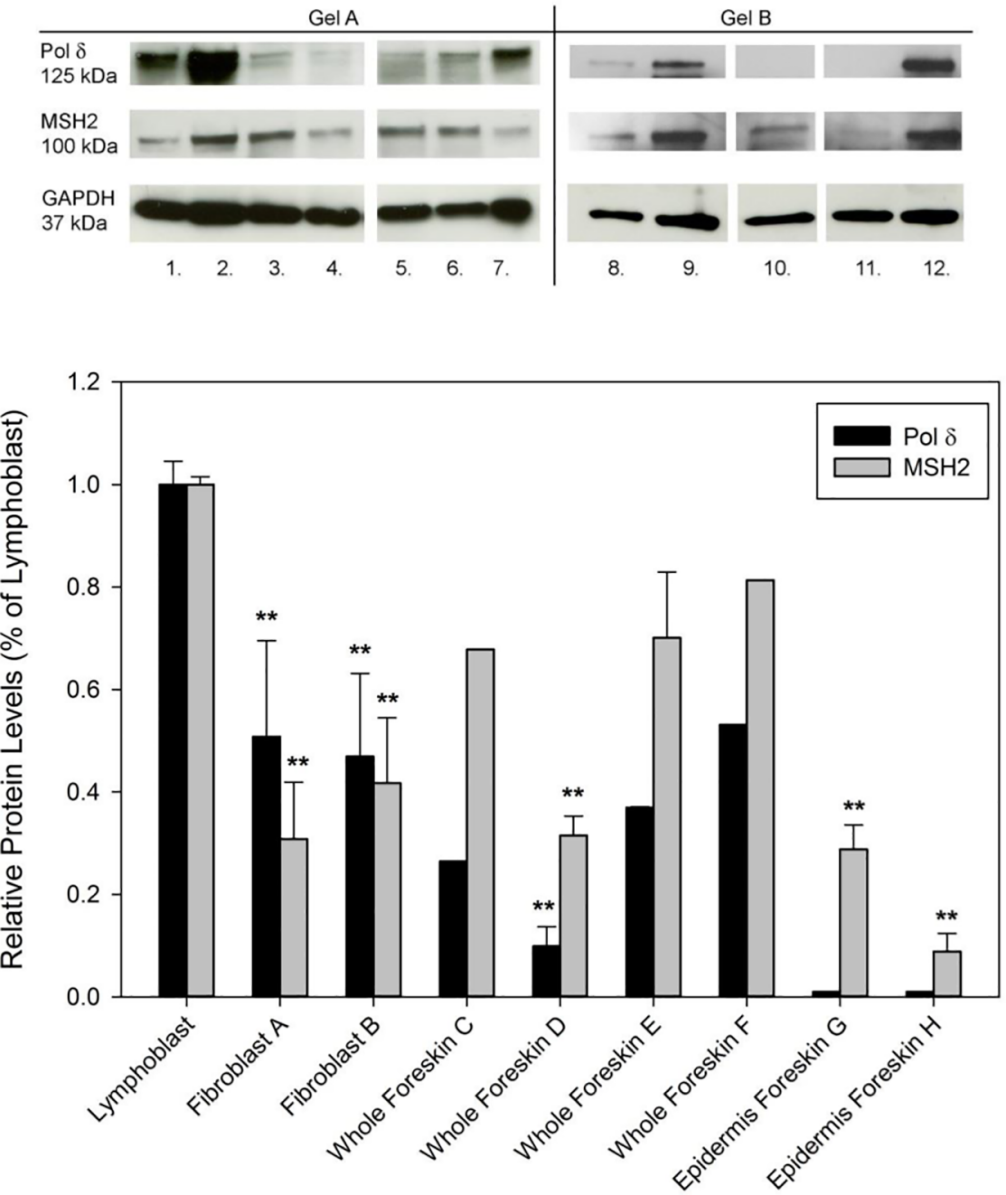
Lower protein levels of MSH2 and polymerase delta in skin compared to blood. A. Immunoblot analysis of whole cell lysates from lymphoblastoid cells (lane 2, 9, 12), cultured skin fibroblasts (lane 1, 7, 8) whole foreskin samples (lanes 3–6) and epidermis isolated from foreskins (lane 10, 11), probed with antibodies against Pol delta and MSH2. GAPDH was used as loading control. Gel A and Gel B are representative immunoblots. B: The levels of the analyzed proteins were normalized to GAPDH and represented as percentage of the corresponding values in lymphoblastoid cells. Bars indicate mean ± SD, **P < 0.005 compared to the lymphoblastoid cells.

How might this mosaicism arise? The finding of these *SLC24A5* sequence variations in the neonatal foreskins suggests that some of them may have originated from spontaneous mutations during embryonic development. Mosaic post-zygotic de novo point mutations were recently identified as a source of genomic variation [37]. An extensively studied yeast model identified spontaneous mutations located at specific DNA sequences [21–23]. Abnormalities in DNA replication polymerases delta or epsilon can result in lagging strand or leading strand mutations with A to G or G to A transitions on one strand [21–23]. These mutations are normally removed by the mismatch repair system which involves several proteins including MSH2. In the yeast system, the mutation rate increased about 10,000-fold in the absence of MSH2 and polymerase delta function. Similarly, human brain tumors from children with inherited mismatch repair deficiency had massive numbers of base substitution mutations [38]. There is evidence that skin cells have lower levels of expression of *MSH2* mRNA than blood cells [http://biogps.org]. We found consistently lower levels of polymerase delta and MSH2 protein in foreskins compared to blood cells (Fig 9). We hypothesize that one possible mechanism to explain the generation of these changes might be that these sequence variations were introduced into progenitor stem cells of the epidermis during embryonic development. They were not removed because of the lower levels of polymerase delta and mismatch repair protein MSH2 in skin compared to blood cells.

### Nature and extent of epidermal mosaicism

We found epidermal mosaicism in *SLC24A5* and six other SNPs located on three different chromosomes (chromosome 15: *MYEF2*, *CTXN2*, *SLC24A5*; chromosome 11: *TYR*; and chromosome 5: *SLC45A2*) (Fig 1 and S8 Fig). However, these changes may be more widespread than we identified. Broader genome wide analyses of these alterations across the genome, and from multiple samples within the same individual would help characterize the extent of these changes. This might be best accomplished with methodology focusing on small samples or single epidermal basal cells.

Interestingly, the most consistent variations we observed in this study involved all possible transitions and transversions, and occurred in exons, introns and sequences outside of genes but were localized at previously identified SNPs (Fig 1 and S8 Fig). Strikingly, Sanger sequencing only identified two alternative nucleotides at each SNP and did not find other nucleotides. While the mechanism of generation of these variations is not understood, we hypothesize that it may be related to the generation of some of the approximately 10 million SNPs in genomic DNA (about 1 every 300 nucleotides) (https://ghr.nlm.nih.gov/primer/genomicresearch/snp).

### Forensics

The SLC24A5 SNP has been proposed as a forensic marker for determining racial identity based on skin DNA found at crime scenes [39, 40]. Our finding of marked epidermal mosaicism would urge great caution in interpretation of forensic data from skin, which might commonly differ from germline sequence.

### Conclusion

Germline sequence stability has been an anchor upon which fundamental biologic, medical and forensic crime scene assumptions have been based. We report an unexpected, dynamic, high frequency of rapidly changing DNA sequence variations (mosaicism) in normal human skin in people of different races. This variation occurs within the epidermis as basal cells divide and move vertically towards the surface and also across the two-dimensional area of the skin surface. The additional variation over time presents a true fourth dimension of mosaicism. This surface variation across races, despite uniform germline (buccal or blood) SNP’s suggests an evolutionary advantage to this SNP mosaicism, possibly as part of an innate cutaneous defense system or genomic decoy to protect people of all races against invading microorganisms [41].

We utilized this variation to identify and map a previously unrecognized alternating patterning of the superficial layers of the epidermis that occurs during epidermal differentiation. While the dynamics of epidermal proliferation have been extensively studied, little is known about the restructuring that occurs at the skin surface during the final stages of terminal differentiation, when the last viable keratinocytes transform into non-viable, but functional, corneocytes. While this surface layer is the most visible and interactive aspect of ourselves, it continues to hold hidden secrets of basic biology and genetics.

## Materials and methods

### Human tissues and cell lines

Formalin fixed, paraffin embedded sections from biopsies of skin surrounding cancers from xeroderma pigmentosum patients were studied. We obtained anonymous foreskins from local hospitals of neonates following circumcision. Foreskins were stored in phosphate-buffered saline (PBS) and kept at 4^o^ C until being transported to NIH. For analysis, we cut each sample into 2 or 3 pieces and placed them in sterilized cell culture dishes. After washing with PBS we gently scraped the surface using a sterile number 15 surgical blade and collected cells for DNA extraction and sequencing (S3 Fig). We isolated DNA from scraped skin surfaces, blood and buccal cells from normal volunteers, and from xeroderma pigmentosum or trichothiodystrophy [15] patients who had been examined at the National Institutes of Health (NIH) Clinical Center under protocols approved by the National Cancer Institute (NCI) Institutional Review Board. Lymphoblastoid cell line (FXP0022) and normal skin fibroblast cell lines (AG13354 and C3PV) were obtained and grown as described previously [42].

### Skin surface scraping

We developed a non-invasive skin surface scraping technique to obtain large numbers of specimens. To obtain DNA from the surface layers of skin we cleaned the skin with 70% isopropyl alcohol swabs and marked five 25x 25 mm areas with ink using a template (S6 Fig). After cleaning the skin surface with an isopropyl alcohol wipe, a sterile number 15 surgical blade (Becton Dickinson, Franklin Lakes, NJ) was also cleaned with alcohol and used to gently scrape the skin surface areas 15 to 20 times. This procedure resulted in no bleeding, erosion, nor visible alteration of the skin, but the small amount of visible stratum corneum on the blade edge was collected for DNA extraction and sequencing. We sampled the outer forearm, inner forearm and/or sun protected buttock skin. The same 5 ink marked areas of the inner forearm were tested repeatedly for 3 months in two subjects.

### Laser capture microdissection of foreskin

We prepared 5-μm PEN membrane-coated slides from formalin fixed, paraffin-embedded foreskin or adult skin tissue blocks. We collected DNA using laser microdissection (Leica LMD 6000, Wetzlar, Germany) with H&E stained slides as described previously [43]. About 300 cells were collected from each sample.

### Enzymatic separation of layers of foreskin

To separate the stratum corneum from the deeper layers of the epidermis, foreskins were injected with 20 μl of 4μg/μl exfoliative toxin A (Toxin Technology, Inc, Sarasota, FL, USA) [20] and incubated for 24 or 48 hours at 37^o^ C (S5 Fig). The epidermis was separated from the dermis by incubation with dispase (BD Biosciences, San Jose, CA) [19] (25 caseinolytic units per foreskin) for 24 hours at 4°C (S5 Fig).

### DNA analysis

We extracted DNA from the scraping or laser capture microdissection (LCM) with QIAamp DNA Micro Kit (QIAGEN, Valencia, CA) or PicoPure DNA Extraction Kit (Life Technologies Grand Island, NY) using protocols supplied by the manufacturer. The primers and PCR conditions listed in S6 Table were employed to amplify the SNP region using Advantage cDNA PCR kit with DNA from skin scraping or from laser capture microdissection. In order to determine the percentage of each allele necessary to be identified, a series of dilutions of each allele was constructed and analyzed (Fig 2A). The Sanger sequencing technology could detect minority alleles that constituted at least 20% of the total DNA (see reconstruction data in Fig 2A). To exclude the possibility that our reagents were contaminated with DNA, we performed multiple control reactions using reagents without added DNA. These reactions were uniformly negative for the PCR-RFLP genotyping, Sanger sequencing, TaqMan™ Real Time PCR and Digital Droplet PCR assays.

### PCR-RFLP genotyping

The forward and reverse primers for PCR amplification of the SNP region (rs1426654) in the *SLC24A5* gene are described in S6 Table. The A>G change creates a new *HhaI* restriction site. DNA was PCR amplified using Advantage cDNA PCR Kit (Clontech Laboratories, Mountain View, CA) and then digested with *HhaI* restriction enzyme from New England BioLabs Inc. (Ipswich, MA) as per their instructions. The PCR products were resolved on a 2% agarose gel. The PCR bands in the gel were visualized with EZ-Vision (Amresco, Solon, OH) and UV light and photographed. The digested DNA showed two bands of 71 and 100 bps and the undigested showed a band of 171 bps (S1 Fig).

### TaqMan Real-Time PCR assay

The rs1426654 genotype was determined using TaqMan™ Real-Time PCR allelic discrimination validated assay with primers and probes specific for the A and G alleles, with TaqMan™ Universal PCR Master Mix (Life Technologies, Grand Island, NY) as per their instructions except the PCR was run on the Bio-Rad iCycler iQ Real-Time PCR detection system (Bio-Rad, Hercules, CA). The *SLC24A5* allelic variants, A and G, were determined by comparing the relative endpoint fluorescence created by the degradation of each fluorescently labelled TaqMan probe (VIC and FAM dye-labeled).

### Digital droplet PCR assay

The *SLC24A5* SNP was detected using digital droplet PCR. Human blood DNA was used as a positive control and water was used as a negative control. *SLC24A5* SNP region was PCR amplified using the primer pair, DNA samples and the conditions as described in S6 Table. The concentration of the PCR product was determined to be about 2 ng/μl using Tape station system 2200 (Agilent Technologies, Santa Clara, CA). The digital droplet PCR assay was performed on a QX200 Droplet Digital PCR System (Bio-Rad, Hercules, CA), consisting of a C1000 Touch Thermalcycler, a QX200 Automated Droplet Generator, and a QX200 Droplet Reader. The PCR reaction mixture (total 22 μL) contained 9.9 μL diluted PCR products (~2100 molecules) and water plus1.1 μL of each primer/probe mix (Assay ID: C_2908190_10, Life Technologies, Grand Island, NY) with VIC (for A) and FAM (for G) fluorophores (20X) and 11 μL of digital droplet PCR Supermix (no dUTP). In brief, the droplet generator divides the samples into 20,000 droplets; these droplets were then transferred into PCR plate for amplification and the droplet readers reads these droplets for A or G nucleotides by providing specific fluorescence signals (VIC for A and FAM for G nucleotides). A total amount of 20 ng of blood DNA was added per well in case of positive controls and the PCR products from DNA extracted from either scraped skin surface or buccal cells from donors were used. The thermal cycling started with 10 min at 95°C, followed by 45 cycles of 94°C for 30 s and 55°C for 60 s. Results were analyzed using Quantasoft v.1.7 software (Bio-Rad).

### Protein lysates

Foreskins of circumcised newborn males were rinsed in PBS and cut into 5x5 mm pieces. For protein isolation of whole foreskins, samples were snap-frozen in liquid nitrogen and cold-processed at 2000 rpm in a Mikro-Dismembrator S (Sartorius, Goettingen, Germany) as described [44].

The epidermis was separated as above. Protein lysates were obtained from powdered whole foreskin samples, epidermis of foreskins, primary fibroblast cell lines and a lymphoblast cell line using cold radioimmunoprecipitation assay buffer [RIPA;50 mM Tris-HCl (pH 8), 150 mM NaCl, 1 mM EDTA, 1% Triton X-100] supplemented with protease inhibitor cocktail (Sigma, P8340). Lysates were incubated on ice for 20 min with vortexing every 4 min, centrifuged at 7000 rpm for 20 min and supernatants quantified with the Bradford method.

### Immunoblot analysis

Proteins samples were run on 4–15% SDS-PAGE gels (Mini-PROTEAN TGX, Biorad, Hercules, CA) and blotted onto nitrocellulose membranes (0.45 μm pore size, Life Technologies, Grand Island, NY). Immunoblotting was performed using Western Breeze Chemiluminescent Western Blot Immunodetection Kit (Invitrogen, Waltham, MA). The following primary antibodies were used: Rabbit anti-MSH2 antibody (sc-22771, Santa Cruz Dallas, TX) 1:500, mouse-anti-polymerase delta (sc-17776, Santa Cruz) 1:250, mouse-anti-GAPDH (MAB374, EMD Millipore, Billerica, MA) 1:7500.

## Supporting information

S1 Fig PCR-RFLP based *SLC24A5* SNP genotyping. The 171 bp PCR fragment of *SLC24A5 DNA* containing only A alleles is resistant to HhaI digestion. However, the presence of G results in HhaI cutting the DNA into two fragments of 100 bp and 71 bp. The gel shows that Donor 1 DNA yields only the 171 bp band before and after Hha1 digestion, indicating the A/A genotype. HhaI digestion of DNA from Donor 2 shows 3 bands (at 171, 100 and 71 bp) indicating that G alleles are present. Hhal digestion of DNA from donor 3 results in only two bands at 100 and 71 bp, indicating the pure G/G genotype. B. Sanger sequencing confirmed the genotype assignments for Donor 1 (A/A), Donor 2 (A/G) and Donor 3 (G/G) indicated by PCR-RFLP digestion.
(PDF)

S2 Fig Laser Capture Microdissection (LCM)and DNA sequencing of neonatal foreskin. A LCM and sequencing of DNA from foreskin W14c revealed *SLC24A5* A/A in the superficial epidermis (including stratum corneum), A/G (60% A and 40%G) in the mid epidermis, A/G (80% A and 20%G) in the epidermal- dermal junction and G/G in the dermis.
(PDF)

S3 Fig Diagram of method for dividing foreskins and enzyme separation of the epidermis from the dermis. The foreskin was cut vertically into 3 pieces and the surface was scraped to remove cells and DNA. The enzyme, dispase, which cleaves fibronectin and type IV collagen [19] was used to separate the epidermis from the dermis and the DNA was sequenced.
(PDF)

S4 Fig Skin surface scraping of neonatal foreskin. A. Cross section of foreskin before scraping. The outer layer of the stratum corneum (*) is present. The basal cells on the lower layer of the epidermis (arrows) are columnar in shape and 5–6 μm in diameter, consistent with earlier studies [32]. B. Cross section of foreskin after scraping. The lowermost layers of the stratum corneum remain (Δ).
(PDF)

S5 Fig Enzymatic separation of layers of foreskin. Exfoliative toxin A (ETA) treatment was used to separate the stratum corneum from the remainder of epidermis [20]. Dispase treatment separated the epidermis from the dermis [19] (See S3 Fig).
(PDF)

S6 Fig Scraping of sites on forearm to obtain skin surface cell DNA. After cleaning the skin surface with 70% isopropyl alcohol, the five 25 x 25 mm areas to be sampled were marked in ink and the surface of the skin was gently scraped using a number 15 sterile scalpel blade. The blade was placed in an Eppendorf tube. The skin surface cells adhering to the blade were collected and the DNA extracted and sequenced.
(PDF)

S7 Fig Length of time between changes in *SCL24A5* SNPs in scraped skin samples. Length of time between changes in *SLC24A5* sequences in samples obtained approximately weekly from skin squares by scraping the skin surface from inner forearm of 2 donors. Each vertical bar represents the number of scraped squares from the inner forearm with allele changes at the indicated time interval (n = 37 pairs of scrapings with changed alleles) (data from Fig 8). The brown line at 14 days indicates the published turnover time for the stratum corneum.
(PDF)

S8 Fig Mosaicism of SNPs on genes in different chromosomes in adult skin scrapings and in neonatal foreskins. Pigmentation related SNPs [24] on different chromosomes (chromosome 15: *MYEF2*, *CTXN2*, *SLC24A5*; chromosome 11: *TYR*; and chromosome 5: *SLC45A2*) were evaluated. A. Skin scrapings from a 70 year old normal female and a 61 year old male with xeroderma pigmentosum were compared to the buccal cells from the same donors. The different DNA sequences are shown in different colors: red and green represent homozygous SNP sequences and yellow is heterozygous. Mosaicism was detected for each of these SNPs. B. Neonatal foreskins from 6 donors were studied. The same foreskins were analyzed as in Fig 3C. Mosaicism was found for *SLC24A5*, *CTXN2* and *SLC45A2*.
(PDF)

S1 Table Subjects and *SLC24A5* SNP germline genotypes studied. A. Race of donors and *SLC24A5* germline genotype. B. Clinical diagnosis of donors and *SLC24A5* germline genotype. C. Gender of donors and *SLC24A5* genotype. [*XP–xeroderma pigmentosum, **TTD–trichothiodystrophy, ***XP/TTD–xeroderma pigmentosum/ trichothiodystrophy complex].
(PDF)

S2 Table Comparison of blood and buccal cell *SLC24A5* SNP germline genotypes in 114 donors. The age, sex, race/ethnicity and clinical phenotype of each donor is indicated. The *SLC24A5* germline genotype was determined in multiple buccal cell samples and in blood. A/A alleles are in green boxes. A/G alleles are in yellow boxes. G/G alleles are in red boxes. The same DNA sequences were identified in each pair for 85 of the donors and only one donor had buccal (G/G) different from blood (A/G) sequences. *C-Caucasian; B- Black; A- Asian; H- Hispanic; M–Mixed. **TTD–trichothiodystrophy; XP- xeroderma pigmentosum; XP/TTD–xeroderma pigmentosum / trichothiodystrophy complex.
(PDF)

S3 Table Taqman Real-time PCR based sequencing of *SLC24A5* A>G SNP (rs1426654) in one donor. The proportion of A/A alleles and G/G alleles was determined from buccal DNA and from DNA isolated from skin scrapings from multiple locations on the arm of donor J. Mixtures of A/A and G/G buccal DNA from different donors were used as control standards. While the buccal DNA from donor J showed 100% A/A, different skin scrapings showed 96 to 100% A/A and 99 to 100% G/G.
(PDF)

S4 Table Variation in *SLC24A5* SNP sequences in 433 skin surface scrapings compared to germline (blood or buccal) cells from 52 donors with 3 or more skin scrapings. *SLC24A5* SNP DNA sequence was determined for donors with 3 or more skin scrapings at different anatomic sites (outer forearm, buttocks, inner forearm) and compared to their germline DNA sequences (from S2 Table). A/A alleles are in green boxes. A/G alleles are in yellow boxes. G/G alleles are in red boxes. We found variations across different anatomic sites and in both sun shielded and sun exposed skin. The anatomic variations were present in donors of different ages, genders, racial groups and clinical phenotypes. *C-Caucasian; B- Black; A- Asian; H- Hispanic; M–Mixed. **TTD–trichothiodystrophy; XP- xeroderma pigmentosum; XP/TTD–xeroderma pigmentosum / trichothiodystrophy complex.
(PDF)

S5 Table Variation in *SLC24A5* SNP in skin surface cell scraping samples. A. Multiple sites of skin scraping at one time for 114 different donors of different genotypes. A total of 598 skin scraping samples were analyzed. Only 31% of the skin scrapings were identical to the germline sequence. One change was found in 57% of the samples and 2 changes in 12%. There was a significantly greater frequency of 2 sequence changes from germline G/G to skin A/A (25%) than from germline A/A to skin G/G (12%) (p = 0.002). B. Multiple sites of skin scraping over 3 months for 2 donors with different germline genotypes. A total of 78 skin scraping samples were analyzed. Only 36% of the skin scraping were identical to the germline sequence. One change was found in 29% of the samples and 2 changes in 35%. There was a significantly greater frequency of 2 sequence changes from germline G/G to skin A/A (71%) than from germline A/A to skin G/G (7%) (p<0.0001).
(PDF)

S6 Table Primers and PCR conditions used in this study. A. Primers used in this study. The forward and reverse primer sequences are listed for the 7 indicated SNPs in the 6 genes. B. PCR conditions used in this study for all of the primers listed.
(PDF)
