# Supplementary figures and images for: Four-dimensional, dynamic mosaicism is a hallmark of normal human skin that permits mapping of the organization and patterning of human epidermis during terminal differentiation
Source: PLoS One. 2018 Jun 13;13(6):e0198011. doi: 10.1371/journal.pone.0198011 (PMC5999106; doi:10.1371/journal.pone.0198011)

A

Donor 1 1 1 2 3 3

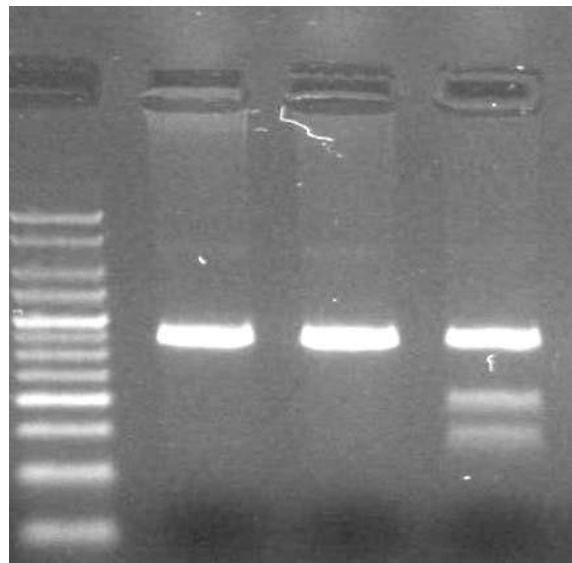

← 171 bp →  
← 100 bp →  
← 71 bp →

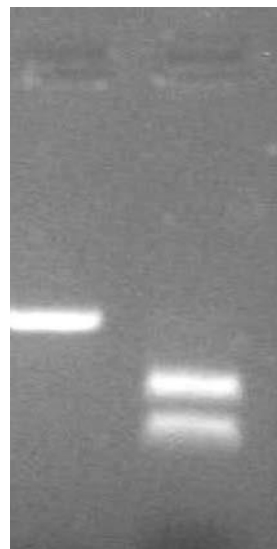

HhaI

-

+

+

-

+

A

171 bp

A

A

171 bp

71 bp

G

100 bp

HhaI

71 bp

G

100 bp

G

B

Donor 1 - A/A

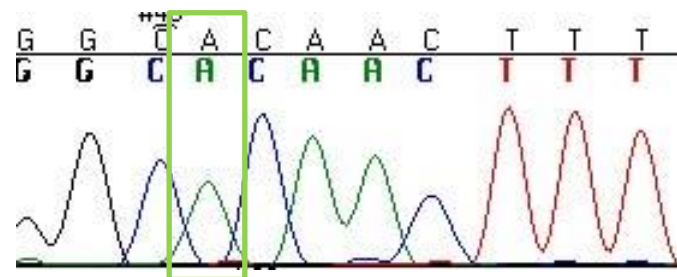

Donor 2 - A/G

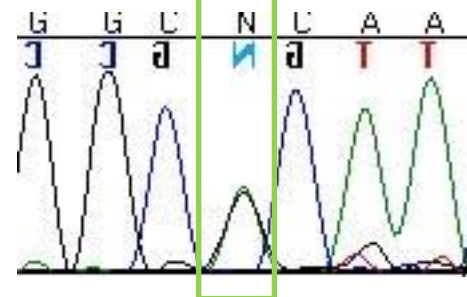

Donor 3 - G/G

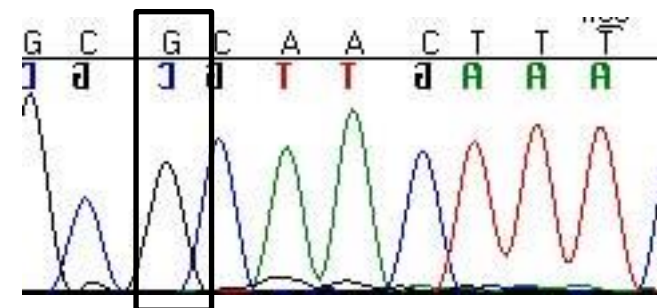

Supplement: S1 Fig — The 171 bp PCR fragment of SLC24A5 DNA containing only A alleles is resistant to HhaI digestion. However, the presence of G results in HhaI cutting the DNA into two fragments of 100 bp and 71 bp. The gel shows that Donor 1 DNA yields only the 171 bp band before and after Hha1 digestion, indicating the A/A genotype. HhaI digestion of DNA from Donor 2 shows 3 bands (at 171, 100 and 71 bp) indicating that G alleles are present. Hhal digestion of DNA from donor 3 results in only two bands at 100 and 71 bp, indicating the pure G/G genotype. B. Sanger sequencing confirmed the genotype assignments for Donor 1 (A/A), Donor 2 (A/G) and Donor 3 (G/G) indicated by PCR-RFLP digestion. (PDF) [file pone.0198011.s001.pdf]

**W14c**

**Superficial**

**A/A 100%**

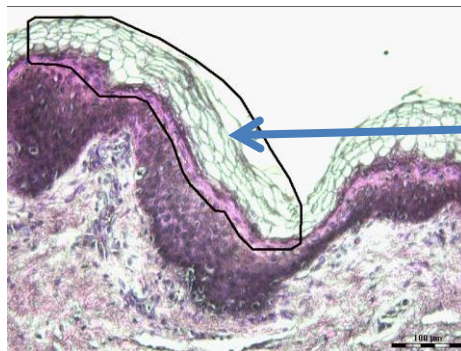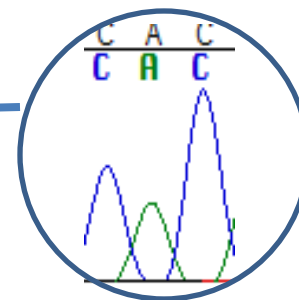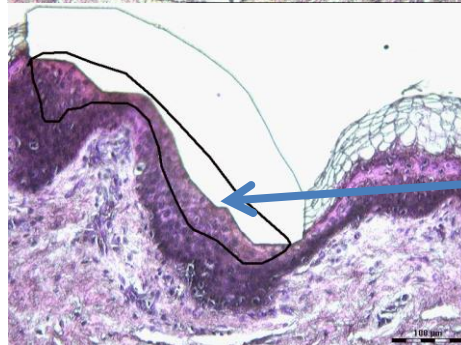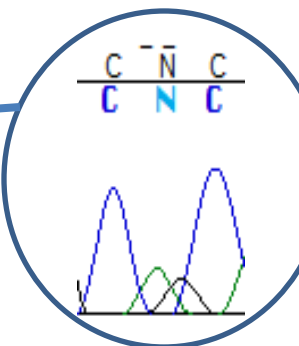

**Mid.  
epidermis**

**A  
60%**

**G  
40%**

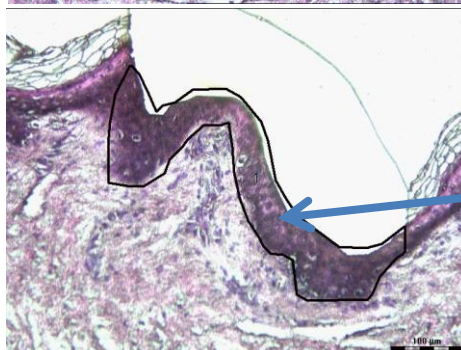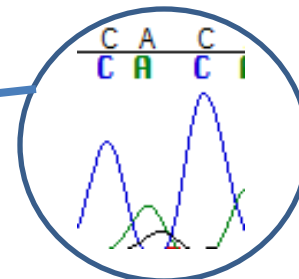

**Junction**

**A  
80%**

**G  
20%**

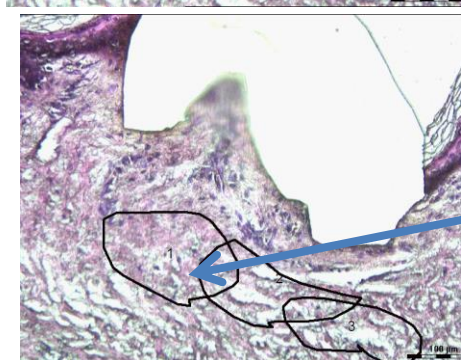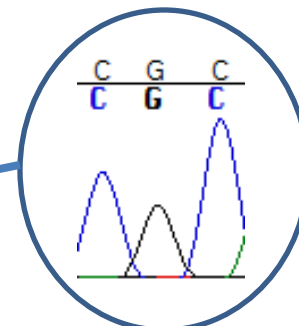

**Dermis**

**G/G 100%**

Supplement: S2 Fig — A LCM and sequencing of DNA from foreskin W14c revealed SLC24A5 A/A in the superficial epidermis (including stratum corneum), A/G (60% A and 40%G) in the mid epidermis, A/G (80% A and 20%G) in the epidermal- dermal junction and G/G in the dermis. (PDF) [file pone.0198011.s002.pdf]

A before scraping

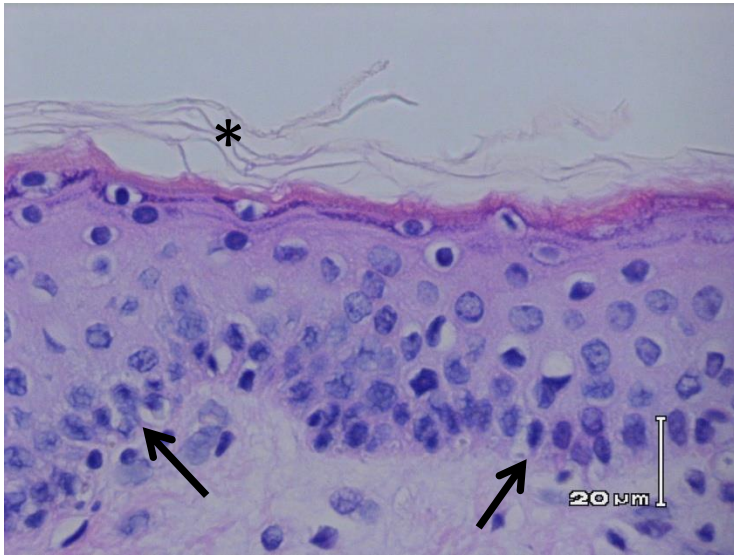

B after scraping

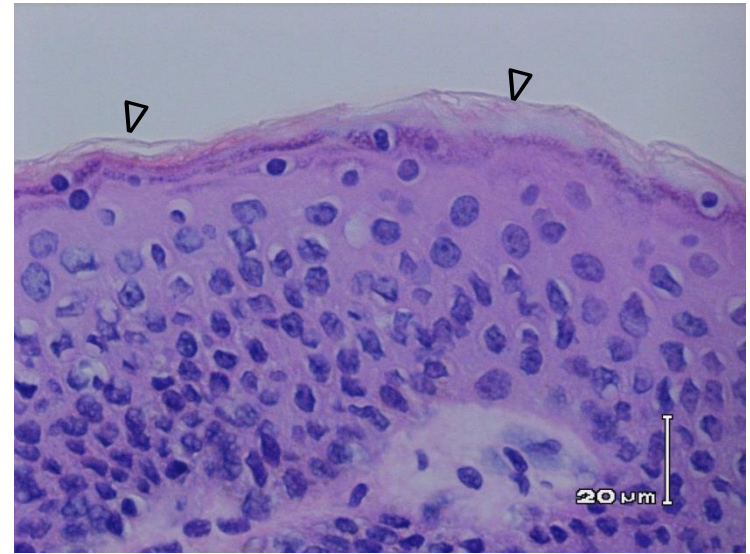

Supplement: S4 Fig — A. Cross section of foreskin before scraping. The outer layer of the stratum corneum (*) is present. The basal cells on the lower layer of the epidermis (arrows) are columnar in shape and 5–6 μm in diameter, consistent with earlier studies [32]. B. Cross section of foreskin after scraping. The lowermost layers of the stratum corneum remain (Δ). (PDF) [file pone.0198011.s004.pdf]

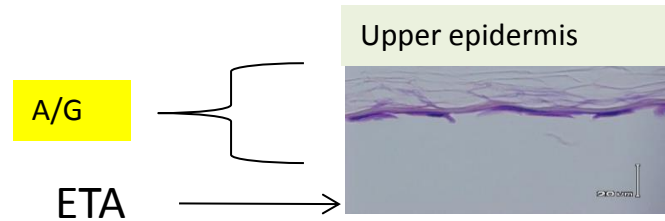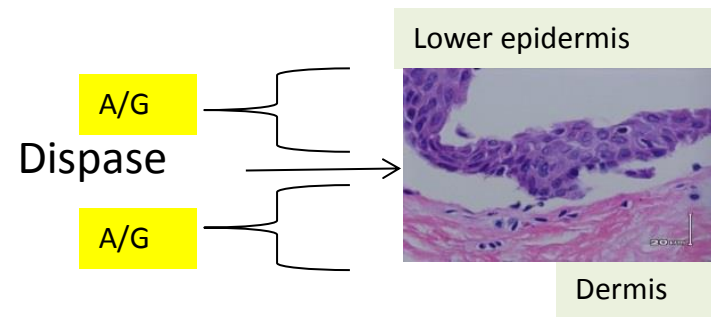

Supplement: S5 Fig — Exfoliative toxin A (ETA) treatment was used to separate the stratum corneum from the remainder of epidermis [20]. Dispase treatment separated the epidermis from the dermis [19] (See S3 Fig). (PDF) [file pone.0198011.s005.pdf]

Length of time between changes in *SLC24A5* sequences  
in scraped skin squares

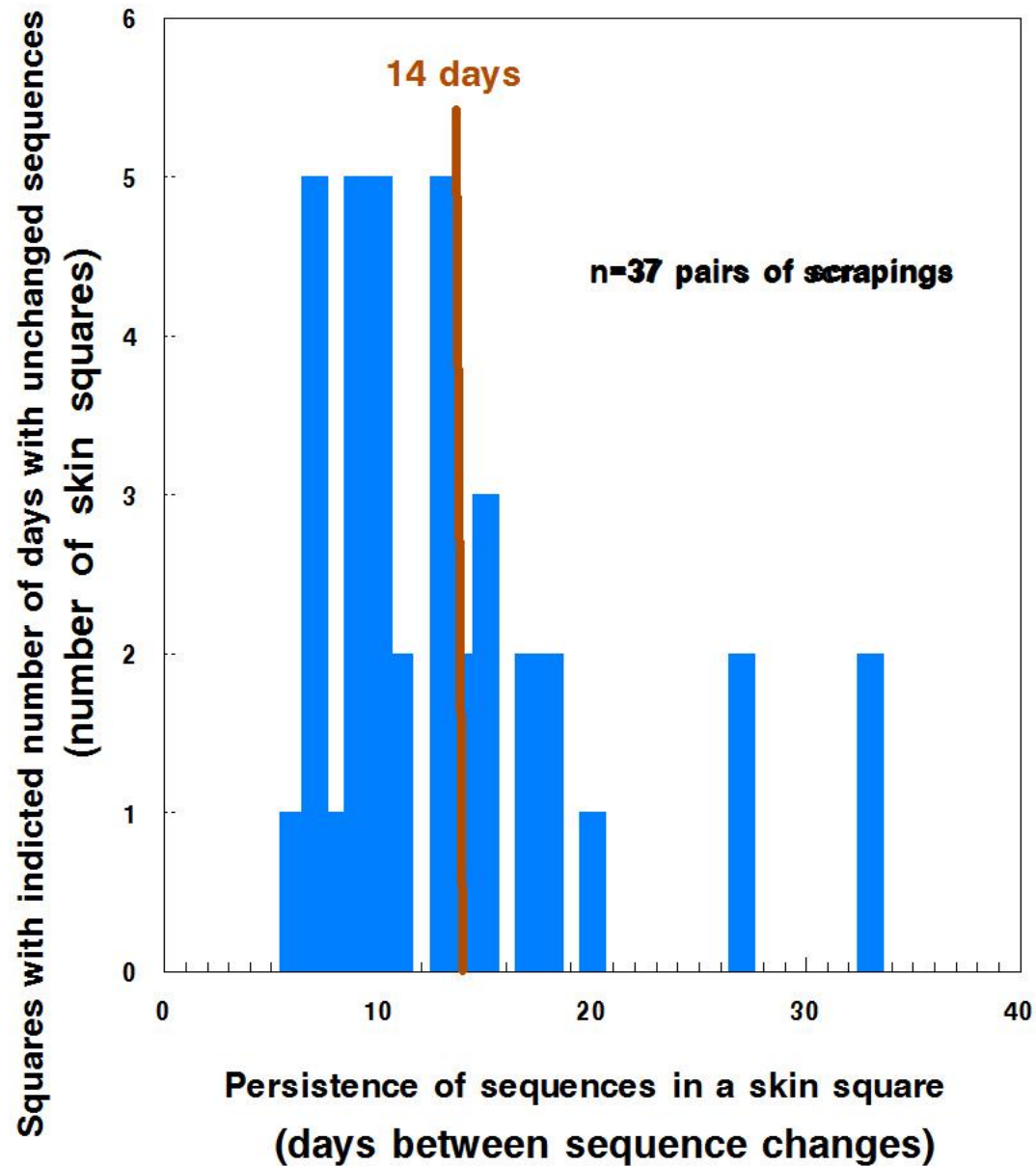

Supplement: S7 Fig — Length of time between changes in SLC24A5 sequences in samples obtained approximately weekly from skin squares by scraping the skin surface from inner forearm of 2 donors. Each vertical bar represents the number of scraped squares from the inner forearm with allele changes at the indicated time interval (n = 37 pairs of scrapings with changed alleles) (data from Fig 8). The brown line at 14 days indicates the published turnover time for the stratum corneum. (PDF) [file pone.0198011.s007.pdf]
