# Supplementary material for: Four-dimensional, dynamic mosaicism is a hallmark of normal human skin that permits mapping of the organization and patterning of human epidermis during terminal differentiation
Source: PLoS One. 2018 Jun 13;13(6):e0198011. doi: 10.1371/journal.pone.0198011 (PMC5999106; doi:10.1371/journal.pone.0198011)
Supplement: S3 Fig — The foreskin was cut vertically into 3 pieces and the surface was scraped to remove cells and DNA. The enzyme, dispase, which cleaves fibronectin and type IV collagen [19] was used to separate the epidermis from the dermis and the DNA was sequenced. (PDF) [file pone.0198011.s003.pdf]

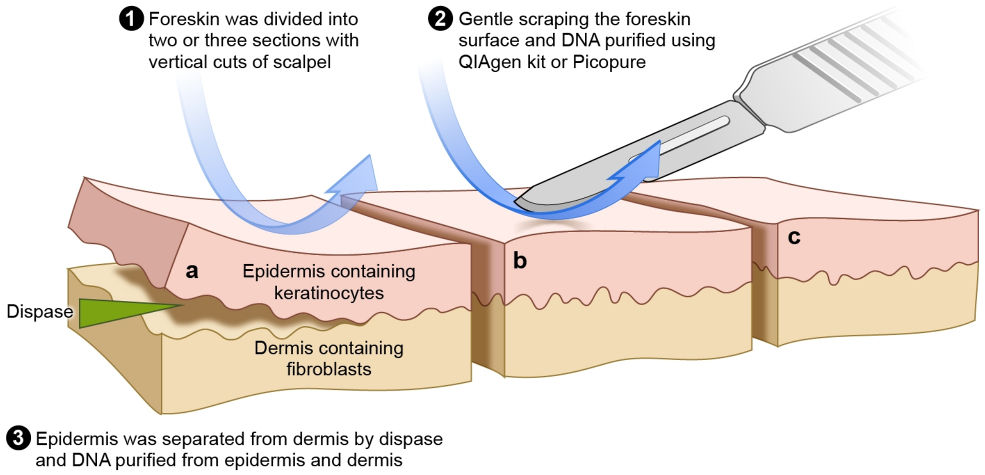

1 Foreskin was divided into two or three sections with vertical cuts of scalpel
2 Gentle scraping the foreskin surface and DNA purified using QIAgen kit or Picopure
a
b
c
Epidermis containing keratinocytes
Dispase
Dermis containing fibroblasts
3 Epidermis was separated from dermis by dispase and DNA purified from epidermis and dermis
