# Supplementary material for: Four-dimensional, dynamic mosaicism is a hallmark of normal human skin that permits mapping of the organization and patterning of human epidermis during terminal differentiation
Source: PLoS One. 2018 Jun 13;13(6):e0198011. doi: 10.1371/journal.pone.0198011 (PMC5999106; doi:10.1371/journal.pone.0198011)
Supplement: S6 Fig — After cleaning the skin surface with 70% isopropyl alcohol, the five 25 x 25 mm areas to be sampled were marked in ink and the surface of the skin was gently scraped using a number 15 sterile scalpel blade. The blade was placed in an Eppendorf tube. The skin surface cells adhering to the blade were collected and the DNA extracted and sequenced. (PDF) [file pone.0198011.s006.pdf]

Wipe skin surface with alcohol

Mark areas to be sampled
using template

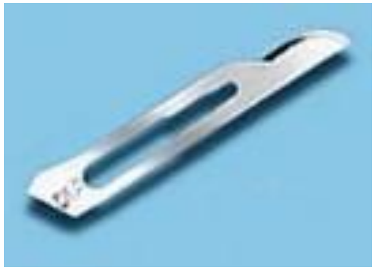

No.15 sterile blade

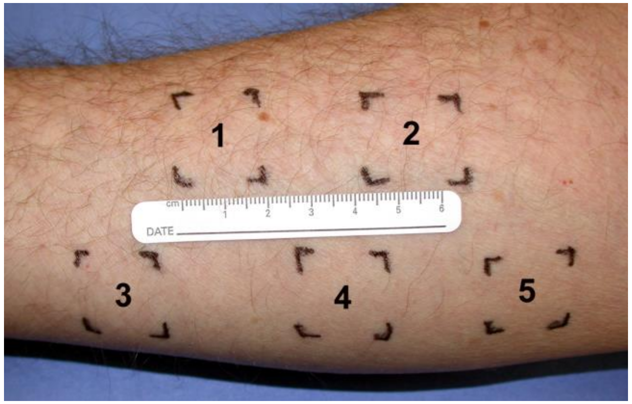

Scraping skin surface gently (15-20 times per site)

Collect skin surface cells
adhering to the blade into
Eppendorf tube.

DNA extracted using QIAamp® DNA Micro Kit or PicoPure® DNA Extraction Kit.
