# Supplementary material for: Four-dimensional, dynamic mosaicism is a hallmark of normal human skin that permits mapping of the organization and patterning of human epidermis during terminal differentiation
Source: PLoS One. 2018 Jun 13;13(6):e0198011. doi: 10.1371/journal.pone.0198011 (PMC5999106; doi:10.1371/journal.pone.0198011)
Supplement: S8 Fig — Pigmentation related SNPs [24] on different chromosomes (chromosome 15: MYEF2, CTXN2, SLC24A5; chromosome 11: TYR; and chromosome 5: SLC45A2) were evaluated. A. Skin scrapings from a 70 year old normal female and a 61 year old male with xeroderma pigmentosum were compared to the buccal cells from the same donors. The different DNA sequences are shown in different colors: red and green represent homozygous SNP sequences and yellow is heterozygous. Mosaicism was detected for each of these SNPs. B. Neonatal foreskins from 6 donors were studied. The same foreskins were analyzed as in Fig 3C. Mosaicism was found for SLC24A5, CTXN2 and SLC45A2. (PDF) [file pone.0198011.s008.pdf]

A- Adult skin

Age (yr)	Sex	Race/ Ethnicity	Clinical Phenotype	Site	46,179,457 Chr 15 Not in gene rs1834640	46,213,776 Chr 15 (SLC24A5) p.Thr111Ala rs1426654	46,258,816 Chr 15 (MYEF2) Upstream rs11070627	46,275,146 Chr 15 (CTXN2) Intron rs12913316	46,282,800 Chr 15 (CTXN2) 3'UTR rs1320052	Chr 11 (TYR) p.Ser192Tyr rs1042602	Chr 5 (SLC45A2) p.Phe374Leu rs16891982
70	F	W	XP mother	Buccal Cells	A/A	A/A	T/T	T/T	T/T	A/A	G/G
				Inner forearm	G/G	G/G	A/T	T/T	-	C/C	G/G
				Outer forearm	-	G/G	A/A	C/T	-	A/C	G/G
61	M	W	XP	Buccal - left	A/G	A/A	T/T	T/T	T/T	A/C	G/G
				Buccal - right	A/G	A/A	T/T	T/T	T/T	A/C	G/G
				Outer forearm 1	-	G/G	A/T	T/T	C/T		C/C
				Outer forearm 2	-	A/G	-	C/T	-	C/C	G/G
				Outer forearm 3	-	G/G	A/T	T/T	-	A/C	C/C
				Outer forearm 4	A/G	A/G	T/T	-	-	A/C	-
				Outer forearm 5	-	G/G	A/A	CC	-	C/C	-

B - Neonatal foreskins

Site	Chr 15 Not in gene rs1834640	Chr 15 (SLC24A5) p.Thr111Ala rs1426654	Chr 15 (MYEF2) Upstream rs11070627	Chr 15 (CTXN2) Intron rs12913316	Chr 15 (CTXN2) 3' UTR rs1320052	Chr 11 (TYR) p.Ser192Tyr rs1042602	Chr 5 (SLC45A2) p.Phe374Leu rs16891982
W1 scraping	A/A	A/G	T/T	C/T	-	C/C	-
W1 epidermis	-	A/A	-	-	-	-	-
W1 dermis	-	A/A	-	-	-	-	-
W2 scraping	A/A	G/G	T/T	C/T	-	C/C	-
W2 epidermis	A/A	A/A	T/T	T/T	T/T	-	G/G
W2 dermis	A/A	A/A	T/T	T/T	-	-	G/G
W3a scraping	A/A	A/A	-	C/T	T/T	A/C	-
W3a epidermis	A/A	A/A	-	TT	T/T	A/C	G/G
W3a dermis	A/A	A/A	-	TT	T/T	A/C	G/G
W3b scraping	A/A	A/A	-	C/T	-	A/C	C/G
W3b epidermis	A/A	A/A	-	T/T	T/T	A/C	G/G
W3b dermis	A/A	A/A	-	T/T	T/T	A/C	G/G
W4a scraping	A/A	A/G	-	T/T	T/T	A/A	G/G
W4a epidermis	A/A	A/A	T/T	T/T	T/T	A/A	G/G
W4a dermis	A/A	A/A	-	T/T	T/T	A/A	G/G
W4b scraping	A/A	A/A	T/T	-	-	A/A	G/G
W4b epidermis	A/A	A/A	T/T	T/T	T/T	A/A	G/G
W4b dermis	A/A	A/A	T/T	T/T	T/T	A/A	G/G
W4c scraping	A/A	A/A	T/T	T/T	-	A/A	G/G
W4c epidermis	A/A	A/A	T/T	T/T	T/T	A/A	G/G
W4c dermis	A/A	A/A	T/T	T/T	T/T	A/A	G/G
B1a scraping	A/A	A/A	-	C/T	T/T	A/C	C/C
B1a epidermis	A/A	A/A	-	T/T	T/T	A/C	C/C
B1a dermis	A/A	A/A	T/T	T/T	T/T	A/C	C/C
Black1b scraping	A/A	A/A	T/T	T/T	T/T	A/C	C/C
Black1b epidermis	A/A	A/A	T/T	T/T	T/T	A/C	C/C
Black1b dermis	A/A	A/A	T/T	T/T	T/T	A/C	C/C
B2a scraping	GG	G/G	T/T	T/T	T/T	-	C/C
B2a epidermis	G/G	G/G	T/T	T/T	T/T	C/C	C/C
B2a dermis	GG	G/G	T/T	T/T	T/T	C/C	C/C
B2b scraping	G/G	A/G	T/T	T/T	-	C/C	C/C
B2b epidermis	G/G	G/G	T/T	T/T	T/T	C/C	C/C
B2b dermis	G/G	G/G	T/T	T/T	T/T	C/C	C/C
B2C scraping	G/G	G/G	T/T	T/T	-	C/C	C/C
B2c epidermis	G/G	G/G	T/T	T/T	-	C/C	C/C
B2c dermis	G/G	G/G	T/T	T/T	T/T	C/C	C/C
