# Supplementary material for: Four-dimensional, dynamic mosaicism is a hallmark of normal human skin that permits mapping of the organization and patterning of human epidermis during terminal differentiation
Source: PLoS One. 2018 Jun 13;13(6):e0198011. doi: 10.1371/journal.pone.0198011 (PMC5999106; doi:10.1371/journal.pone.0198011)
Supplement: S1 Table — A. Race of donors and SLC24A5 germline genotype. B. Clinical diagnosis of donors and SLC24A5 germline genotype. C. Gender of donors and SLC24A5 genotype. [*XP–xeroderma pigmentosum, **TTD–trichothiodystrophy, ***XP/TTD–xeroderma pigmentosum/ trichothiodystrophy complex]. (PDF) [file pone.0198011.s009.pdf]

S1 Table - Subjects and *SLC24A5* SNP germline genotypes studied

A

RACE	DONORS (number)	SLC24A5 SNP GERMLINE GENOTYPE - BUCCAL CELLS AND/OR BLOOD		
		**A/A**	**A/G**	**G/G**
Caucasian	59	59	0	0
Black	21	2	8	11
Asian	19	2	3	14
Hispanic	5	0	4	1
mixed	10	3	5	2
total	114	66	20	28

B

CLINICAL DIAGNOSIS	DONORS (number)	SLC24A5 SNP GERMLINE GENOTYPE - BUCCAL CELLS AND/OR BLOOD		
		**A/A**	**A/G**	**G/G**
Normal	86	44	16	26
XP*	15	11	3	1
TTD**	8	6	1	1
XP/TTD***	5	5	0	0
total	114	66	20	28

C

GENDER	DONORS (number)	SLC24A5 SNP GERMLINE GENOTYPE - BUCCAL CELLS AND/OR BLOOD		
		**A/A**	**A/G**	**G/G**
Female	62	39	13	10
Male	52	27	7	18
total	114	66	20	28

*XP- xeroderma pigmentosum

**TTD - trichothiodystrophy

***XP/TTD - xeroderma pigmentosum/ trichiothiodystophy complex
