# Supplementary material for: Four-dimensional, dynamic mosaicism is a hallmark of normal human skin that permits mapping of the organization and patterning of human epidermis during terminal differentiation
Source: PLoS One. 2018 Jun 13;13(6):e0198011. doi: 10.1371/journal.pone.0198011 (PMC5999106; doi:10.1371/journal.pone.0198011)
Supplement: S2 Table — The age, sex, race/ethnicity and clinical phenotype of each donor is indicated. The SLC24A5 germline genotype was determined in multiple buccal cell samples and in blood. A/A alleles are in green boxes. A/G alleles are in yellow boxes. G/G alleles are in red boxes. The same DNA sequences were identified in each pair for 85 of the donors and only one donor had buccal (G/G) different from blood (A/G) sequences. *C-Caucasian; B- Black; A- Asian; H- Hispanic; M–Mixed. **TTD–trichothiodystrophy; XP- xeroderma pigmentosum; XP/TTD–xeroderma pigmentosum / trichothiodystrophy complex. (PDF) [file pone.0198011.s010.pdf]

S2 Table - Comparison of blood and buccal cell *SLC24A5* SNP germline genotypes in 114 donors

DONORS (n=114)				*SLC24A5* SNP GERMLINE GENOTYPE			
Age	Sex	Race/ Ethnicity*	Clinical Phenotype**	BUCCAL CELLS (sample 1) n=111	BUCCAL CELLS (sample 2) n=38	BUCCAL CELLS (sample 3) n=11	BLOOD n=76
1.5	M	C	TTD	A/A	A/A		A/A
3	M	C	Normal	A/A	A/A		A/A
5	F	C	TTD	A/A	A/A		A/A
7	F	C	TTD	A/A	A/A		A/A
7	F	C	TTD	A/A	A/A		A/A
7	M	C	TTD	A/A	A/A		A/A
7	M	C	TTD				A/A
10	A	C	Normal	A/A			A/A
11	F	C	XP	A/A			A/A
13	M	M	XP				A/A
13	F	C	XP/TTD	A/A			A/A
13	F	c	Normal	A/A			A/A
14	F	M	Normal	A/A			A/A
15	F	C	Normal	A/A			A/A
17	M	B	Normal	A/A	A/A		A/A
17	M	C	XP	A/A	A/A		A/A
20	F	C	XP/TTD	A/A			A/A
20	F	C	Normal	A/A			A/A
23	F	C	Normal	A/A	A/A		A/A
23	M	A	XP	A/A			A/A
24	F	C	Normal	A/A			A/A
25	F	C	Normal	A/A	A/A		A/A
26	F	C	Normal	A/A			
27	M	C	Normal	A/A			
27	F	C	XP	A/A	A/A		A/A
27	M	C	Normal	A/A			A/A
28	F	A	Normal	A/A			A/A
29	F	C	XP	A/A			A/A
30	F	C	Normal	A/A			A/A
30	F	C	Normal	A/A			
31	F	C	XP/TTD	A/A			A/A
31	F	C	Normal	A/A			A/A
33	M	C	Normal	A/A			A/A
34	F	C	XP	A/A			A/A
34	M	C	XP	A/A			A/A
35	M	C	Normal				A/A
35	F	C	Normal	A/A			A/A
37	M	C	Normal	A/A			A/A
39	M	C	Normal	A/A	A/A		
39	F	C	Normal	A/A	A/A	A/A	A/A
41	M	C	Normal	A/A	A/A	A/A	A/A
42	F	C	XP	A/A			A/A
43	M	C	Normal	A/A			A/A
45	F	C	Normal	A/A			A/A
45	F	C	Normal	A/A	A/A	A/A	A/A
46	F	B	Normal	A/A	A/A		A/A
46	F	C	Normal	A/A			A/A
47	F	C	Normal	A/A			A/A
50	M	C	Normal	A/A			A/A
51	M	C	Normal	A/A			A/A
52	F	C	XP/TTD	A/A			
52	F	C	XP	A/A			A/A
54	F	C	Normal	A/A	A/A		A/A
54	M	C	Normal	A/A			A/A
56	F	C	Normal	A/A			A/A
56	M	C	Normal	A/A			A/A
60	F	C	Normal	A/A			A/A
60	M	C	XP	A/A			A/A
61	M	C	XP	A/A	A/A	A/A	A/A
62	F	C	Normal	A/A			A/A
63	M	C	Normal	A/A	A/A	A/A	A/A
64	M	C	Normal	A/A			A/A
67	F	C	Normal	A/A			A/A
68	M	C	Normal	A/A	A/A		A/A
70	F	C	Normal	A/A			A/A
72	M	C	Normal	A/A			A/A
3	F	M	TTD	A/G	A/G		A/G
16	F	B	XP	A/G	A/G		A/G
17	F	H	Normal	A/G	A/G	A/G	A/G
17	M	B	XP	A/G	A/G		A/G
20	F	A	Normal	A/G			
21	F	B	Normal	A/G	A/G	A/G	
21	F	M	Normal	A/G	A/G		
22	M	B	XP	A/G	A/G		A/G
23	M	M	Normal	A/G	A/G		
24	M	B	Normal	A/G	A/G		
25	F	B	Normal	A/G			
25	F	B	Normal	A/G			
26	F	M	Normal	A/G			A/G
26	M	M	Normal	A/G			
41	F	A	Normal	A/G	A/G		
41	F	H	Normal	A/G	A/G	A/G	
41	F	H	Normal	A/G	A/G	A/G	A/G
45	M	A	Normal	A/G	A/G	A/G	
50	F	M	Normal	A/G			A/G
55	M	B	Normal	A/G	A/G		A/G
3	M	H	Normal	G/G	G/G		
3	F	M	TTD	G/G			A/G
4	M	H	Normal	G/G	G/G		
11	F	B	Normal	G/G			G/G
13	M	B	XP	G/G			G/G
20	F	B	Normal	G/G			
22	F	A	Normal	G/G			
23	F	B	Normal	G/G	G/G		
26	M	B	Normal	G/G			
26	M	B	Normal	G/G			
26	F	H	Normal	G/G			
27	M	B	Normal	G/G			G/G
29	F	A	Normal	G/G			
29	M	A	Normal	G/G			
30	F	A	Normal	G/G			
32	M	A	Normal	G/G	G/G	G/G	
32	F	B	Normal	G/G			
32	M	A	Normal	G/G			
33	M	A	Normal	G/G			
33	M	A	Normal	G/G			
35	M	B	Normal	G/G			G/G
36	M	A	Normal	G/G			
37	M	A	Normal	G/G			
38	M	A	Normal	G/G			
38	M	A	Normal	G/G			
39	M	B	Normal	G/G			G/G
43	F	A	Normal	G/G			
61	M	A	Normal	G/G			

*C-Caucasian; B- Black; A- Asian; H- Hispanic; M- mixed
**TTD- trichothiodystrophy; XP - xeroderma pigmentosum;
XP/TTD - xeroderma pigmentosum/ trichothiodystrophy complex
