# Supplementary material for: Four-dimensional, dynamic mosaicism is a hallmark of normal human skin that permits mapping of the organization and patterning of human epidermis during terminal differentiation
Source: PLoS One. 2018 Jun 13;13(6):e0198011. doi: 10.1371/journal.pone.0198011 (PMC5999106; doi:10.1371/journal.pone.0198011)
Supplement: S3 Table — The proportion of A/A alleles and G/G alleles was determined from buccal DNA and from DNA isolated from skin scrapings from multiple locations on the arm of donor J. Mixtures of A/A and G/G buccal DNA from different donors were used as control standards. While the buccal DNA from donor J showed 100% A/A, different skin scrapings showed 96 to 100% A/A and 99 to 100% G/G. (PDF) [file pone.0198011.s011.pdf]

S3 Table - Taqman Real-time PCR based sequencing of SLC24A5 A>G SNP (rs1426654) in one donor

Samples	A/A SQ mean	%	G/G SQ mean	%	DECLARED SEQUENCE
Donor J buccal DNA	5.72E+03	100%	0	0%	**A/A**
Donor J DNA from inner arm - square 1	3.23E+01	96%	1.31E+00	4%	**A/A**
Donor J DNA from outer arm - square 1	2.50E+03	100%	0	0%	**A/A**
Donor J DNA from inner arm - square 2	0	0%	1.93E+05	100%	**G/G**
Donor J DNA from outer arm - square 2	2.97E+05	100%	1.19E-02	0%	**A/A**
Donor J DNA from inner arm - square 3	0.018	0%	6.55E+02	100%	**G/G**
Donor J DNA from outer arm - square 3	4.82	1%	6.74E+02	99%	**G/G**
Donor J DNA from inner arm - square 4	0.203	0%	7.85E+02	100%	**G/G**
Donor J DNA from outer arm - square 4	2.53	0%	7.35E+02	100%	**G/G**
Standard with A/A (100% A/A + 0% G/G buccal DNA)	3.77E+04	100%	0	0%	**A/A**
Standard with A/G (75% A/A + 25% G/G buccal DNA)	5.57E+04	66%	2.86E+04	34%	**A/G**
Standard with A/G (50% A/A + 50% G/G buccal DNA)	2.41E+04	56%	1.93E+04	44%	**A/G**
Standard with A/G (25% A/A + 75% G/G buccal DNA)	7.72E+02	0%	1.62E+05	100%	**G/G**
Standard with G/G (0% A/A + 100% G/G buccal DNA)	0	0%	1.29E+05	100%	**G/G**
