# Supplementary material for: Four-dimensional, dynamic mosaicism is a hallmark of normal human skin that permits mapping of the organization and patterning of human epidermis during terminal differentiation
Source: PLoS One. 2018 Jun 13;13(6):e0198011. doi: 10.1371/journal.pone.0198011 (PMC5999106; doi:10.1371/journal.pone.0198011)
Supplement: S4 Table — SLC24A5 SNP DNA sequence was determined for donors with 3 or more skin scrapings at different anatomic sites (outer forearm, buttocks, inner forearm) and compared to their germline DNA sequences (from S2 Table). A/A alleles are in green boxes. A/G alleles are in yellow boxes. G/G alleles are in red boxes. We found variations across different anatomic sites and in both sun shielded and sun exposed skin. The anatomic variations were present in donors of different ages, genders, racial groups and clinical phenotypes. *C-Caucasian; B- Black; A- Asian; H- Hispanic; M–Mixed. **TTD–trichothiodystrophy; XP- xeroderma pigmentosum; XP/TTD–xeroderma pigmentosum / trichothiodystrophy complex. (PDF) [file pone.0198011.s012.pdf]

S4 Table - Variation in *SLC24A5* SNP sequences in 433 skin surface scrapings compared to germline (blood or buccal) cells from 52 donors with 3 or more skln scrapings

DONORS				SLC24A5 GENOTYPE			SKIN SURFACE SCRAPING SEQUENCES														
							OUTER FOREARM					BUTTOCKS					INNER FOREARM				
Age	Sex	Race/ Ethnicity*	Clinical Phenotype**	BUCCAL CELLS (sample 1)	BUCCAL CELLS (sample 2)	BLOOD	Outer 1	Outer 2	Outer 3	Outer 4	Outer 5	Shield 1	Shield 2	Shield 3	Shield 4	Shield 5	Inner 1	Inner 2	Inner 3	Inner 4	Inner 5
1.5	M	C	TTD	A/A	A/A	A/A		A/A	A/A	A/G			G/G	A/G	G/G	A/G					
3	M	C	Normal	A/A	A/A	A/A		A/G	A/G	A/A	A/G	A/G	A/G	A/A	A/G	A/G					
7	F	C	TTD	A/A	A/A	A/A	A/A	G/G				A/G	A/G		G/G	A/G					
11	F	C	XP	A/A		A/A	A/G	A/A	A/A	G/G	A/G	A/A	A/G	A/A	A/A	A/A	A/A	A/A	A/G	A/A	A/G
13	M	M	XP			A/A			A/G	A/G							A/G	A/G	A/G	A/G	A/G
13	F	C	XP/TTD	A/A		A/A	A/G										A/A				
14	F	M	Normal	A/A		A/A	A/G	A/A	A/G	A/A	A/A						A/G	A/G	A/G	A/G	A/G
17	M	B	Normal	A/A	A/A	A/A	A/G	A/A	A/G	A/G	A/A	A/A	A/A	A/G	A/A	A/A					
20	F	C	XP/TTD	A/A		A/A	A/G										A/A				
23	F	C	Normal	A/A	A/A	A/A			G/G	G/G	A/G		G/G								
25	F	C	Normal	A/A	A/A	A/A	A/G	A/G	A/A	A/G	A/G	A/G	A/G	A/G	A/G	A/G					
27	M	C	Normal	A/A			A/A	A/G	A/A	A/G	A/G						A/G	A/G	A/G	A/A	A/A
27	F	C	XP	A/A	A/A	A/A	A/G	A/A	A/G	A/G		A/A	A/G	A/G	A/G	A/G					
31	F	C	XP/TTD	A/A		A/A	A/G										A/A				
39	M	C	Normal	A/A	A/A		A/G		A/A	A/A	A/A	A/A	A/G	A/A	A/A	A/A					
39	F	C	Normal	A/A	A/A	A/A	A/G	A/G	A/G	A/G	A/G	A/G	A/G	A/G	G/G	A/A					
41	M	C	Normal	A/A	A/A	A/A	A/A	A/A	A/A	A/G	A/G	A/A	G/G	A/A	A/A	G/G					
43	M	C	Normal	A/A		A/A	A/A	A/G	A/A	A/A	G/G						A/A	A/A	A/A	A/G	A/A
45	F	C	Normal	A/A		A/A	G/G	A/A	A/A	A/A	A/A						A/G	A/G	A/A	A/G	A/A
45	F	C	Normal	A/A	A/A	A/A	G/G	A/G	A/G	A/G			A/G	A/G(G is Bigger)	A/G	G/G					
46	F	B	Normal	A/A	A/A	A/A	A/G	A/G	A/G	A/G	A/A	A/A		A/G	A/A	A/A	A/G				
46	F	C	Normal	A/A		A/A	A/A										A/G				
51	M	C	Normal	A/A		A/A	A/A	A/G		A/G	A/A						A/A	A/G	A/G	A/G	A/A
54	F	C	Normal	A/A	A/A	A/A	A/G	A/G	G/G	A/G		A/G	A/G		A/G	A/G					
61	M	C	XP	A/A	A/A	A/A	G/G	A/G	G/G	A/G	G/G	A/G	A/G	G/G	A/G	A/G	A/A				
63	M	C	Normal	A/A	A/A	A/A	A/G	A/G	A/A	A/G	A/G						A/A	A/G	A/G	A/G	A/A
67	F	C	Normal	A/A		A/A		A/G	A/G	A/G		A/G	A/G		A/G	A/A					
68	M	C	Normal	A/A	A/A	A/A	A/G	A/G	A/G	A/G	A/G	A/G	A/G	A/G	A/G	A/G					
3	F	M	TTD	A/G	A/G	A/G	A/G	A/G	A/G	A/G	A/G										
16	F	B	XP	A/G	A/G	A/G	A/A	G/G	A/A	A/A	A/G	A/A	A/A								
17	F	H	Normal	A/G	A/G	A/G		A/G	A/G		G/G	A/A		A/A	A/G	A/G					
17	M	B	XP	A/G	A/G	A/G				G/G	G/G				G/G						
20	F	A	Normal	A/G			A/G	A/A	A/G	A/A	A/A	A/A	A/A	A/A	A/A	A/A					
21	F	B	Normal	A/G	A/G		A/G	A/G	A/G	A/A	A/G						A/G	A/G	A/G	A/G	A/G
21	F	M	Normal	A/G	A/G		A/A	A/A	A/G	A/A	A/A	A/A	G/G	A/A	A/A	A/A					
22	M	B	XP	A/G	A/G	A/G				G/G	A/A			G/G		A/A					
23	M	C	Normal	A/G	A/G		A/A	A/A	A/G	A/A		A/A	A/G	A/A	A/A	A/A					
24	M	B	Normal	A/G	A/G			A/A	A/A	A/A	A/G	A/G	A/G	G/G	A/A	A/A					
25	F	B	Normal	A/G			A/G	A/G	G/G	A/G	A/G						A/G	A/G	A/A	A/G	A/A
41	F	A	Normal	A/G	A/G		A/G	A/G	A/G	A/G	A/G						A/G	A/G	A/G	A/G	A/G
41	F	H	Normal	A/G	A/G		A/A			A/A	A/G	G/G	A/A	A/A		A/A					
41	F	H	Normal	A/G	A/G	A/G	A/G			G/G	A/A	G/G	G/G	A/A	A/A						
50	F	M	Normal	A/G		A/G	G/G	A/G	A/A	A/G	A/G						A/G	A/G	A/G	A/G	A/G
55	M	B	Normal	A/G	A/G	A/G	A/A	A/A	A/G	A/G	A/G	A/A	A/A	A/A		A/G					
3	M	H	Normal	G/G	G/G		A/A	A/G	G/G	G/G	G/G	A/A	A/A	A/G	A/G	A/A					
4	M	H	Normal	G/G	G/G		A/G	A/A	A/G	A/A	A/A	G/G	A/A	A/A	A/G	A/A					
20	F	B	Normal	G/G			A/A	A/A	A/G	G/G	A/A	A/G	A/G	G/G	A/G	A/G					
23	F	B	Normal	G/G	G/G		A/G	A/G	A/A	A/G	A/G	A/G	A/A	A/G	A/G	A/A					
26	M	B	Normal	G/G			A/G	A/G	A/G	G/G	A/G						A/G	A/G	A/G	A/A	A/G
26	M	B	Normal	G/G			A/G	A/A	A/A	A/G							A/G	A/G	A/G	A/G	A/G
32	M	A	Normal	G/G	G/G		A/G	A/G	G/G	A/G	A/G						A/G	A/A	A/G	A/G	A/G
39	M	B	Normal	G/G		G/G	G/G	A/G	A/G	A/G	A/G	A/G	A/G	A/G	A/G	A/G					

*C-Caucasian; B- Black; A- Asian; H- Hispanic; M- mixed

**TTD- trichothiodystrophy; XP - xeroderma pigmentosum; XP/TTD - xeroderma pigmentosum/ trichothiodystrophy complex
