# Supplementary material for: Four-dimensional, dynamic mosaicism is a hallmark of normal human skin that permits mapping of the organization and patterning of human epidermis during terminal differentiation
Source: PLoS One. 2018 Jun 13;13(6):e0198011. doi: 10.1371/journal.pone.0198011 (PMC5999106; doi:10.1371/journal.pone.0198011)
Supplement: S5 Table — A. Multiple sites of skin scraping at one time for 114 different donors of different genotypes. A total of 598 skin scraping samples were analyzed. Only 31% of the skin scrapings were identical to the germline sequence. One change was found in 57% of the samples and 2 changes in 12%. There was a significantly greater frequency of 2 sequence changes from germline G/G to skin A/A (25%) than from germline A/A to skin G/G (12%) (p = 0.002). B. Multiple sites of skin scraping over 3 months for 2 donors with different germline genotypes. A total of 78 skin scraping samples were analyzed. Only 36% of the skin scraping were identical to the germline sequence. One change was found in 29% of the samples and 2 changes in 35%. There was a significantly greater frequency of 2 sequence changes from germline G/G to skin A/A (71%) than from germline A/A to skin G/G (7%) (p<0.0001). (PDF) [file pone.0198011.s013.pdf]

# S5 Table - Variation in SLC24A5 SNP in skin surface cell scraping samples

## A. 114 different donors - multiple sites - one sampling time each

| **Donor genotype (buccal cells or blood)** | **Number of donors** | **(% of donors)** | Total (number of skin samples analyzed for each donor genotype) | | SNP sequence changes per skin sample (number of samples analyzed) (% of skin samples for each donor genotype) **no change** | | **1** | | **2** | | | Samples/ donor |
|---|---|---|---|---|---|---|---|---|---|---|---|---|
| **A/A** | 66 | 58% | 341 | 100% | 107 | 31% | 193 | 57% | 41 | 12% | ¶ | 5.2 |
| **A/G** | 20 | 17% | 135 | 100% | 61* | 45% | 74 | 55% | not detectable | | ** | 6.8 |
| **G/G** | 28 | 25% | 122 | 100% | 20 | 16% | 72 | 59% | 30 | 25% | ¶ | 4.4 |
| **Totals** | 114 | 100% | 598 | 100% | 188 | 31% | 339 | 57% | 71 | 12% | | 5.2 |

¶ p=0.002

*some of these may be 2 changes

**not detectable because two changes of A/G are identical to no change

## B. Two donors - multiple samplings of same sites during 3 months

| **Donor genotype (buccal cells or blood)** | **Number of donors** | **(% of donors)** | Total (number of skin samples analyzed for each donor genotype) | | SNP sequence changes per skin sample (number of samples analyzed) (% of skin samples for each donor genotype) **no change** | | **1** | | **2** | | Samples/ donor |
|---|---|---|---|---|---|---|---|---|---|---|---|
| **A/A** | 1 | 50% | 44 | 100% | 26 | 59% | 15 | 34% | 3 | 7% | 44.0 |
| **G/G** | 1 | 50% | 34 | 100% | 2 | 6% | 8 | 24% | 24 | 71% | 34.0 |
| **Totals** | 2 | 100% | 78 | 100% | 28 | 36% | 23 | 29% | 27 | 35% | 39.0 |
