# Supplementary material for: Four-dimensional, dynamic mosaicism is a hallmark of normal human skin that permits mapping of the organization and patterning of human epidermis during terminal differentiation
Source: PLoS One. 2018 Jun 13;13(6):e0198011. doi: 10.1371/journal.pone.0198011 (PMC5999106; doi:10.1371/journal.pone.0198011)
Supplement: S6 Table — A. Primers used in this study. The forward and reverse primer sequences are listed for the 7 indicated SNPs in the 6 genes. B. PCR conditions used in this study for all of the primers listed. (PDF) [file pone.0198011.s014.pdf]

S6 Table - Primers and PCR conditions used

A. Primers used in this study

Primer name [SNP] (gene)	Sequence(5'-3') Forward	Sequence(5'-3') Reverse
rs1426654 [A/G](SLC24A5)	tttcactttattaggcataacaatca	aaaaatcacactgagtaagcaagaa
rs1834640 [A/G]	tgcattaatagcccccctttg	tgcagcagctccagctataa
rs11070627 [A/T](MYEF2)	tccatgaagaagcaaacacg	tttttcggtagagggtaggaaa
rs12913316 [C/T](CTXN2)	tccaacctacctttccaacg	atcacccattgagggatcag
rs1320052[C/T] (CTNX2)	ggcatggttactgctcaggt	ccaactggggtacagagagc
rs1042602 [A/C] (TYR)	atggccaaatgaaaaatgga	ctatgccaaggcagaaaagc
rs16891982 [C/G] (SLC45A2)	tccaagttgtgctagaccag	cctcaacagcctccaatctc

B. PCR conditions

Temperature (℃)	Time (minute)	Cycles
95	3 minutes	x 1
94	30 seconds	
67	30 seconds	
-0.5 ℃ per cycle		x15
72	40 seconds	
94	30 seconds	
58	30 seconds	x30
72	40 seconds	
72	9 minutes	x 1
15	forever	
